# Pre-Impregnated Powder Coatings with a Thermoplastic Matrix for Use in Fibre-Reinforced Polymer Sandwich Composites: Manufacturing, Mechanical Testing and Finite Element Modelling

**DOI:** 10.3390/ma19183972

**Published:** 2026-09-18

**Authors:** Przemysław Golewski, Michał Budka

**Affiliations:** 1 Group of Solid State Mechanics, Department of Roads and Bridges, Faculty of Civil Engineering and Architecture, Lublin University of Technology, Nadbystrzycka 40 Str., 20-618 Lublin, Poland; 2 Laboratory of Civil Engineering, Faculty of Civil Engineering and Architecture, Lublin University of Technology, Nadbystrzycka 40 Str., 20-618 Lublin, Poland; m.budka@pollub.pl

**Keywords:** pre-impregnated coating, digital image correlation, sandwich composite, CadQuery, representative volume element, tensile test, three-point bending test

## Abstract

This study presents a manufacturing route for pre-impregnated functional powder coatings (PCs) intended for fibre-reinforced polymer (FRP) composites, including vacuum-infused sandwich structures. PCs comprising powder, thermoplastic nonwoven and twill glass fabric were produced by a two-stage heat-pressing process. Ceramic, mineral and metallic powders were investigated. Uniaxial tensile tests with digital image correlation and three-point bending tests of PC-integrated sandwich composites were supported by finite element analyses of representative volume elements. Powder layers were parametrically generated in CadQuery for F24, F30 and F36 grains with ellipsoidal or sharp-edged geometries. The powder and thermoplastic layers markedly affected tensile stiffness and deformation. Apparent Young’s moduli ranged from 3.8 to 8.1 GPa, compared with 11.7–14.3 GPa for reference specimens. Manufacturing-induced curvature and an atypical transverse response were attributed to the asymmetric layered architecture. All sandwich variants reached similar maximum bending loads of 0.93–0.96 kN, while absorbed energy ranged from 7.2 to 9.1 J. RVE simulations showed that grain size affected local stress, strain and displacement fields. The method enables repeatable manufacture of thin, modular functional layers and their integration with composites without applying loose powder during moulding.

## 1. Introduction

Fibre-reinforced polymer composites are now widely used in structures where low weight, high specific strength and stiffness, corrosion resistance, and the ability to tailor material properties through appropriate selection of the matrix, fibres, and stacking sequence are important. These materials are used in aerospace, automotive, energy and marine applications. Their use in civil engineering is also increasing [1], including glass fibre-reinforced polymer (GFRP) bars for concrete reinforcement [2], carbon fibre-reinforced polymer (CFRP) strips and sheets for strengthening existing structures [3], pultruded load-bearing profiles [4], and girders manufactured by vacuum infusion [5]. In these applications, component durability is often determined not only by mechanical properties but also by the surface’s environmental resistance.

Despite the favourable service properties of FRP composites, their surfaces remain susceptible to ultraviolet radiation, moisture, chemicals, elevated temperatures, flame, rain, particle erosion, icing, impact, and scratching. The polymer matrix is particularly vulnerable; its degradation may lead to microcracking, fibre exposure, weakening of the fibre–matrix interface and the initiation of delamination. Fire exposure is a particularly important limitation for GFRP sandwich structures. Recent fire-resistance tests on vacuum-infused GFRP sandwich panels have shown that both the core material and the panel architecture influence their thermomechanical response, and that passive fire-protection systems can substantially improve fire endurance [6]. Consequently, various surface-modification [7] and coating-deposition methods [8,9] are being developed.

Organic coatings, including polyurethane, polyurea, epoxy and silicone systems, constitute one of the largest groups. Zhang et al. [10] produced a femtosecond-laser-textured polyurethane coating on GFRP, obtaining a superhydrophobic surface with anti-icing and self-cleaning properties. On wind turbine blades, polyurethane coatings are primarily used to mitigate leading-edge erosion. Pathak et al. [11] demonstrated that the properties of such coatings could be improved using cellulose microparticles and microfibres. At the same time, modelling of fatigue damage in multilayer systems applied to laminate surfaces indicates that repeated raindrop impacts may cause the protective layer to delaminate from the intermediate layer [12]. Thin multilayer Ti/TiN coatings deposited by physical vapour deposition (PVD) are also being considered for erosion protection, with the optimum coating thickness depending on whether particle or rain erosion is the dominant load [13]. Polyurea coatings, in turn, may reduce back-face deformation in ultra-high-molecular-weight polyethylene (UHMWPE) laminates subjected to ballistic loading, with their effectiveness depending, among other factors, on the coating’s position relative to the impact direction [14].

Coatings on composites may also provide electrical and fire-protection functions. Das et al. [15] used a conductive polyaniline-based coating to protect CFRP and GFRP composites against lightning-strike damage, demonstrating that its effectiveness depended on coating thickness and substrate type. Intumescent coatings and layers containing inorganic flame-retardant additives are being developed for construction and marine applications. Floch et al. [16] developed a coating based on poly(vinyl alcohol), ammonium polyphosphate and sepiolite nanofillers, deposited on glass fabric and intended to protect GFRP laminates. Another approach involves films containing graphene nanoplatelets, which reduce the temperature and the damaged area of CFRP exposed to intense thermal radiation by promoting heat conduction and dissipation [17].

A distinctive class of protective systems comprises ceramic thermal barrier coatings (TBCs). These coatings are used primarily on hot components of aircraft engines and gas turbines [18,19,20,21]. The TBC concept may also be transferred to fibre-reinforced polymer substrates, but this requires solutions to problems arising from the low thermal resistance of the matrix, mismatched coefficients of thermal expansion and the difficulty of obtaining a durable ceramic–polymer bond. Previous studies have demonstrated, among other approaches, the protection of CFRP laminates using Al_2_O_3_ fibre mats integrated with the composite during curing [22], as well as PMC/TBC systems operating at elevated temperatures and high strain rates [21].

Recent years have seen marked progress in the direct deposition of ceramic and metallic coatings onto CFRP composites [23,24]. Kim et al. [25] developed an Al_2_O_3_ coating that limited heating of the composite substrate during exposure to a flame at 500–700 °C. A subsequent study reported a bilayer 7% yttria-stabilised zirconia (7YSZ) coating with controlled porosity, produced by flame spraying with polyether ether ketone (PEEK) particles as a pore-forming agent [26]. Semmler et al. [27] compared the properties of plasma-sprayed 7YSZ, aluminium titanate, cordierite and mullite coatings on cyanate-ester-matrix CFRP, while more recent work deposited porous yttrium aluminium garnet (YAG) coatings directly onto carbon fabric before the vacuum-assisted resin transfer moulding (VARTM) process [28]. This approach limits the direct effect of spraying on the cured matrix and facilitates integration of the ceramic layer with the laminate. Coatings are also being developed to thermally protect CFRP components used in unmanned aerial vehicles, including hybrid metal–TBC–CFRP systems and TiO_2_/graphene bilayer coatings [25,29,30].

An alternative group of solutions comprises layers containing ceramic, metallic or mineral powder grains or particles bound in a polymer matrix [31,32]. The powder type can be selected based on the required function, such as resistance to erosion, local impact, elevated temperature, or electrical conductivity. A drawback of this approach is that the powder is spread directly over the mould surface. To overcome this limitation, the concept of a pre-impregnated coating (PC) was developed as a self-supporting semi-finished product that can be prepared prior to manufacturing the composite component [33]. The resulting sheets can be cut to shape, laid on gently curved surfaces and integrated with sandwich composites during vacuum infusion. The tests confirmed that a durable coating-to-laminate bond could be obtained while maintaining coating integrity under substantial bending deformation.

The present work extends the concept introduced in [33] through a substantially modified PC architecture and a new numerical modelling framework. In the previous design, both the functional and backing layers were powder-based and bonded to a thermoplastic nonwoven. In the present configuration, the powder layer is integrated with a glass-fabric backing layer, while an optional second thermoplastic nonwoven is introduced on the opposite side of the powder layer to enclose the grains and limit resin infiltration during vacuum infusion. Dedicated reference specimens with different arrangements of the glass fabric and thermoplastic nonwoven were also introduced to examine the influence of matrix distribution and fabric impregnation on the mechanical response. Coating behaviour was evaluated in uniaxial tensile tests using DIC, from which strain distributions, failure force, absorbed energy, apparent Young’s modulus and apparent Poisson’s ratio were determined. Three-point bending tests were then performed on sandwich composites integrated with PCs. The study also presents the development of a parametric algorithm in CadQuery for generating three-dimensional grain layers with specified grit size, shape, orientation and random spatial arrangement. On this basis, geometric models of powder layers were created for different FEPA grades and two characteristic grain shapes. Two levels of representative modelling were prepared: a PC model comprising the fabric, thermoplastic layer, and individual grains; and a sandwich-composite model that additionally includes the GFRP laminate, core, and polymer matrix. Numerical modelling plays an important role in analysing the mechanical response of laminated and sandwich composites, particularly due to the strong contrast between the properties of the face sheets and the core. Recent layerwise approaches have demonstrated the importance of accurately representing through-thickness displacement and stress fields in the bending analysis of such structures [34]. The numerical analyses aimed to determine the effects of grain size and geometry on local stress, strain, and displacement distributions and to provide a basis for future extensions that incorporate material nonlinearity, grain fracture, matrix damage, and delamination.

## 2. Materials and Methods

The principal materials used to manufacture the PCs were powders of different grit sizes, a thermoplastic nonwoven and glass fabric. Five powder types were used; the order listed below corresponds to the numbering of the laboratory specimen batches:

1. Green silicon carbide (F220);
2. Glauconite (F60);
3. Stainless-steel grit (DELTA 050);
4. Quartz sand (F80);
5. Chromium electrocorundum (F36).

**Figure 1:**
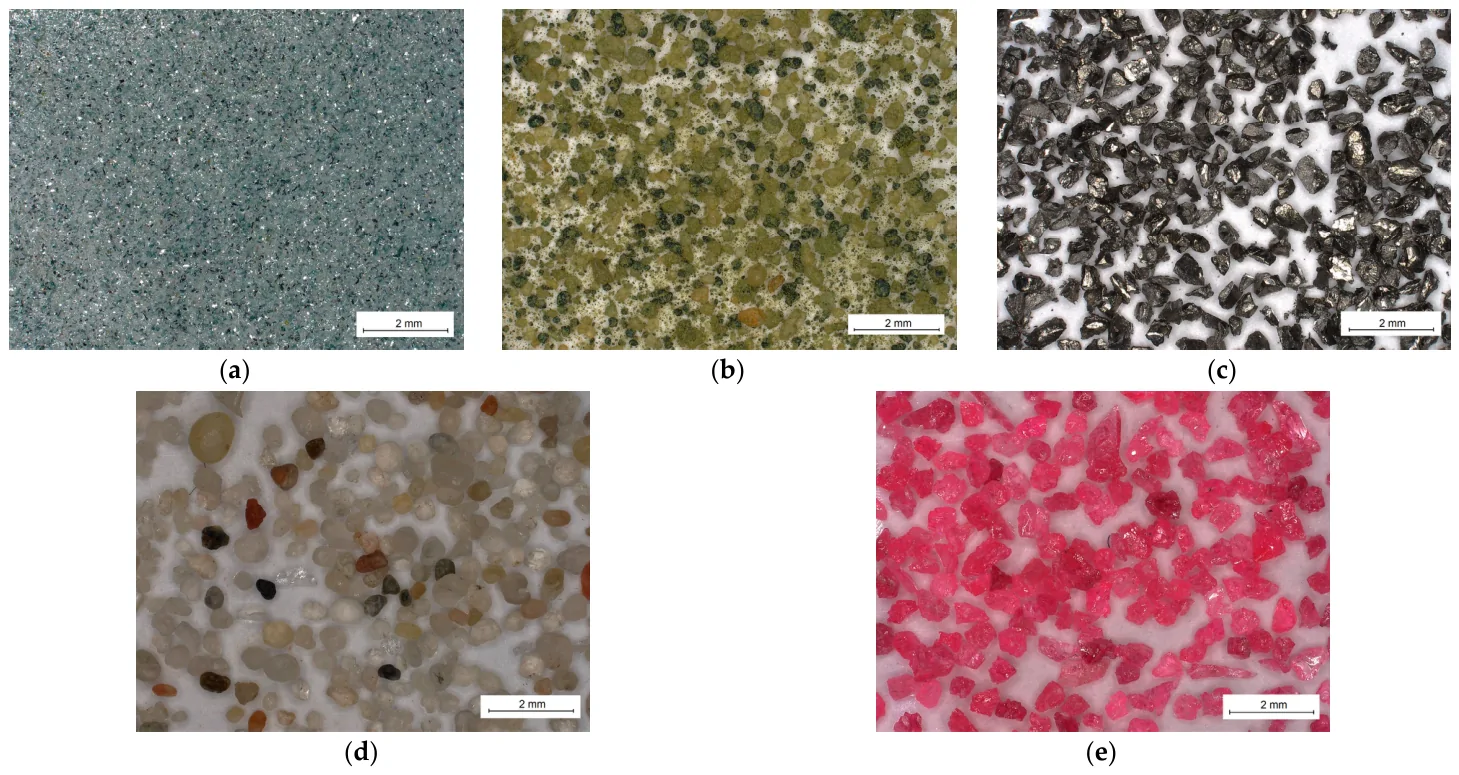
Representative digital-microscope images of the powder materials used in the pre-impregnated coatings: (**a**) green silicon carbide F220, (**b**) glauconite F60, (**c**) stainless-steel grit DELTA 050, (**d**) quartz sand F80, and (**e**) chromium electrocorundum F36. Magnification: ×30.

Representative microscope images of the five powder materials are shown in Figure 1. Clear differences in grain morphology can be observed, ranging from predominantly rounded and subrounded quartz-sand particles to strongly angular and sharp-edged stainless-steel and chromium-electrocorundum grains. Additional information on the origin and basic properties of the powders was reported previously in [33].

The particle dimensions were additionally quantified from calibrated digital-microscope images (Table 1). Four magnifications were acquired for each powder; however, because these represented different magnifications of the same material rather than independent particle populations, one magnification providing an appropriate balance between particle count and spatial resolution was selected for quantitative analysis. The projected area, *A*, of each segmented grain was determined and converted to an equivalent circular diameter (Equation (1)):

(1) $$d_{eq}=2\sqrt{\frac{A}{\pi }}$$

Particles intersecting the image boundaries and regions in which individual grains could not be reliably separated were excluded. At least 98 grains were retained for each powder. The resulting number-based particle-size distributions are reported as the 10th, 50th and 90th percentiles (D_10_, D_50_ and D_90_, respectively). The reported values therefore represent two-dimensional, image-based particle-size distributions and should not be interpreted as equivalent to mass-based sieve or laser-diffraction distributions.

The copolyester thermoplastic nonwoven used to bind the powder to the glass-fabric backing layer (BL) had a melting range of 193–200 °C and an areal density of 79 g/m^2^. It is an innovative product supplied by TMBK Partners Sp. z o.o. (Warsaw, Poland). The final constituent was a 280 g/m^2^ twill-weave glass fabric supplied by SP-TEX Sp. z o.o. (Czechowice-Dziedzice, Poland).

A flexible 3 mm-thick Soric SF core supplied by Lantor (Veenendaal, the Netherlands) was used to manufacture the sandwich composite. It was placed between three plies of 280 g/m^2^ twill-weave glass-fabric reinforcement on each side. The resulting lay-up was 3 × 0°/Soric core/3 × 0°. The composite was manufactured by vacuum infusion using IP2 polyester resin with an MEKP catalyst (Easy Composites, Stoke-on-Trent, UK).

**Figure 2:**
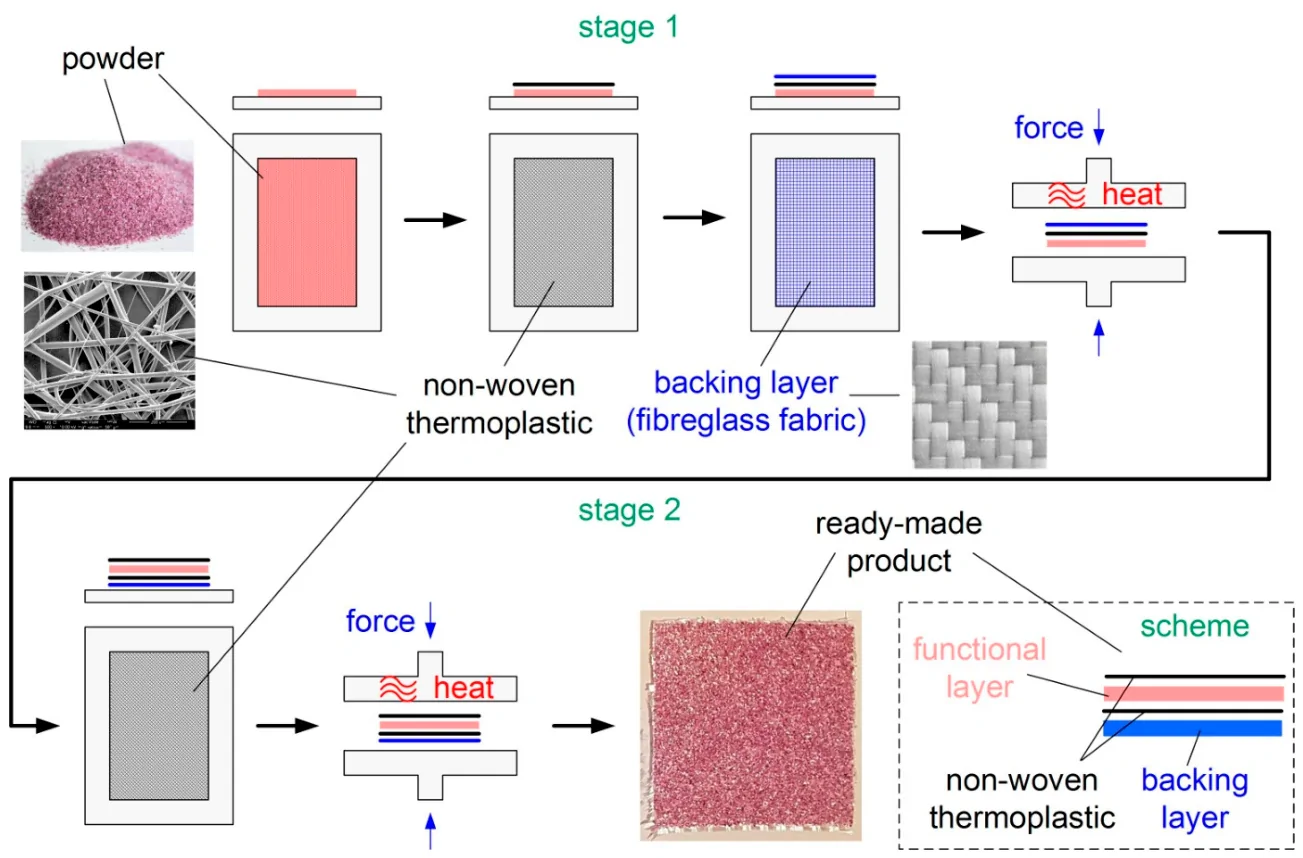
Manufacturing process for the pre-impregnated coatings.

The PCs were manufactured using the same equipment as in [33]: a purpose-built device for spreading the powder layer and a REV 3S Transmatic heat-transfer press (Lazzate, Italy). Although the equipment was unchanged, the PC manufacturing procedure differed and is shown schematically in Figure 2.

**Figure 3:**
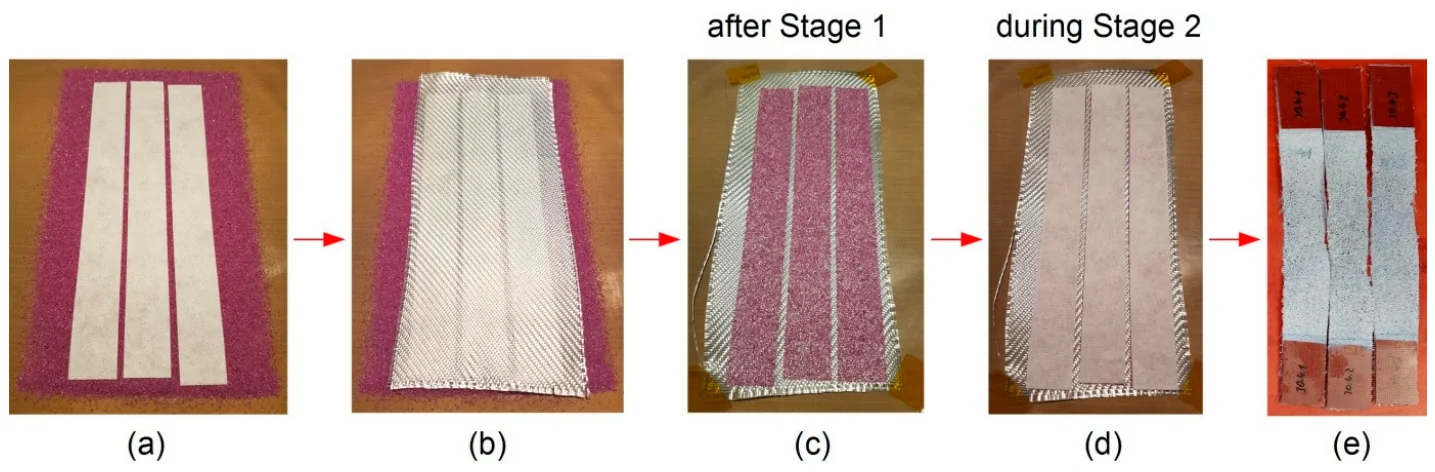
The process of preparing specimens for uniaxial tensile testing: (**a**) thermoplastic nonwoven strips placed on the powder layer; (**b**) placement of the glass-fabric backing layer; (**c**) pre-impregnated coating after the first heat-bonding stage; (**d**) placement of the second thermoplastic nonwoven layer before the second heat-bonding stage; (**e**) finished tensile specimens with bonded end tabs.

The first stage began by spreading a uniformly thick powder layer over the bed of the heat-transfer press. Numerical control of the rotational speed of the spreading roller and the movement of the feeder enabled precise dosing of the grains. The thermoplastic nonwoven was then cut to the required specimen shape. In this study, the dimensions were 50 mm × 50 mm for the bonding trials, 30 mm × 250 mm for the specimens shown in Figure 3a, and 200 mm × 300 mm for the PC-integrated sandwich-composite panels produced by vacuum infusion. The shape, however, may be selected freely according to the product to be coated.

The thermoplastic nonwoven was then covered with a glass-fabric backing layer (Figure 3b). The stack was heat-bonded in the press for 5 min, during which the nonwoven melted and flowed partly between the fabric fibres and partly between the powder grains. Preliminary processing trials are therefore required to ensure that the nonwoven’s areal density is neither too high nor too low. In the former case, the glass fabric may become fully impregnated; in the latter, the powder grains may not consolidate into a coherent layer. After the sheet was removed from the press, excess powder was recovered for use in the next cycle.

The second stage involved enclosing the PC. The product obtained in the first stage (Figure 3c) was positioned with its backing layer in contact with the lower surface of the press. A second thermoplastic nonwoven layer and a release film were then placed over the grains (Figure 3d). The stack was heat-bonded again for 5 min. The final PC, therefore, comprised two principal parts: the backing layer (BL) and the functional layer (FL).

**Figure 4:**
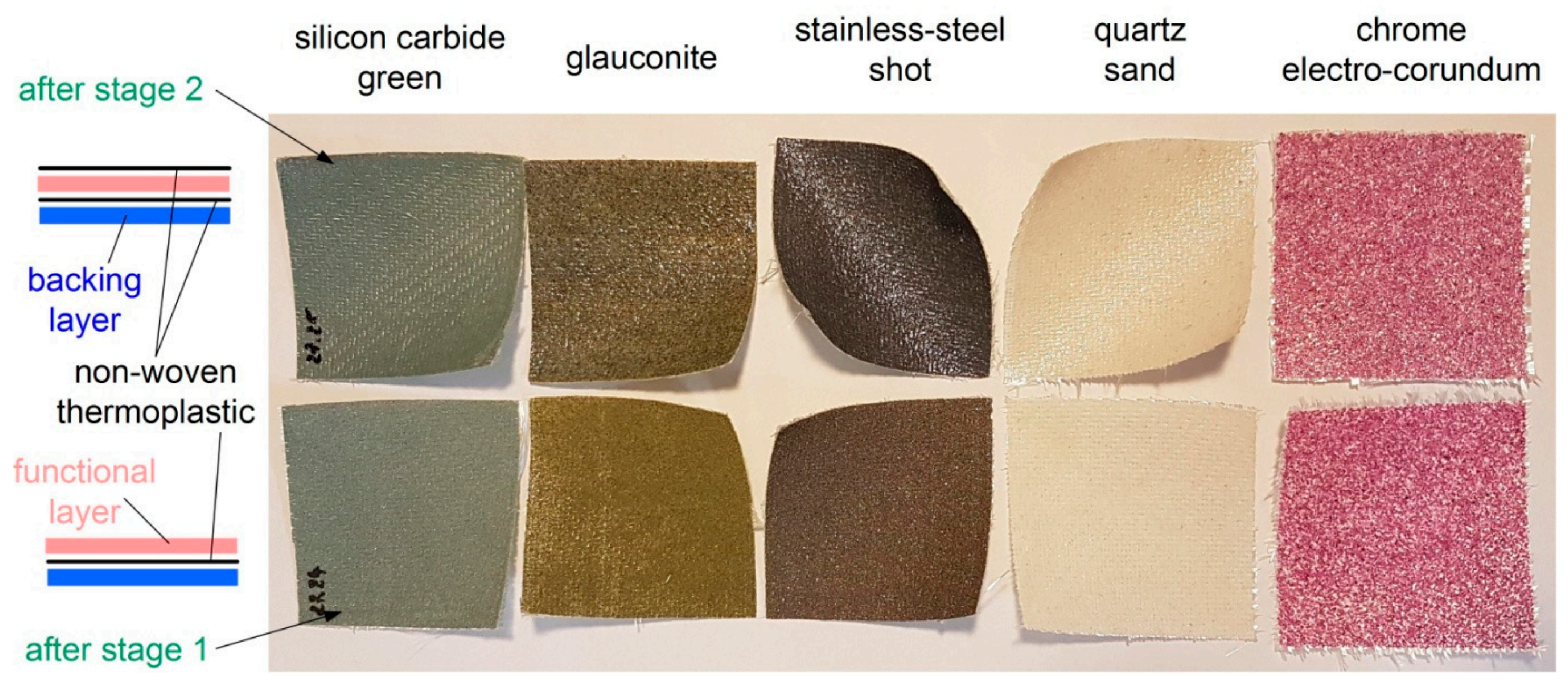
Examples of PCs at different stages of manufacture.

Enclosing the powder layer permanently traps it between thermoplastic layers, preventing infiltration by liquid resin during vacuum infusion. PCs subjected to the enclosing stage, however, are stiffer and exhibit greater initial distortion after manufacture, as illustrated in Figure 4 for all analysed materials. PCs without the enclosing layer are less stiff and can therefore conform more readily to curved surfaces. It should nevertheless be recognised that during vacuum infusion, their powder layer will be fully saturated with liquid resin.

**Figure 5:**
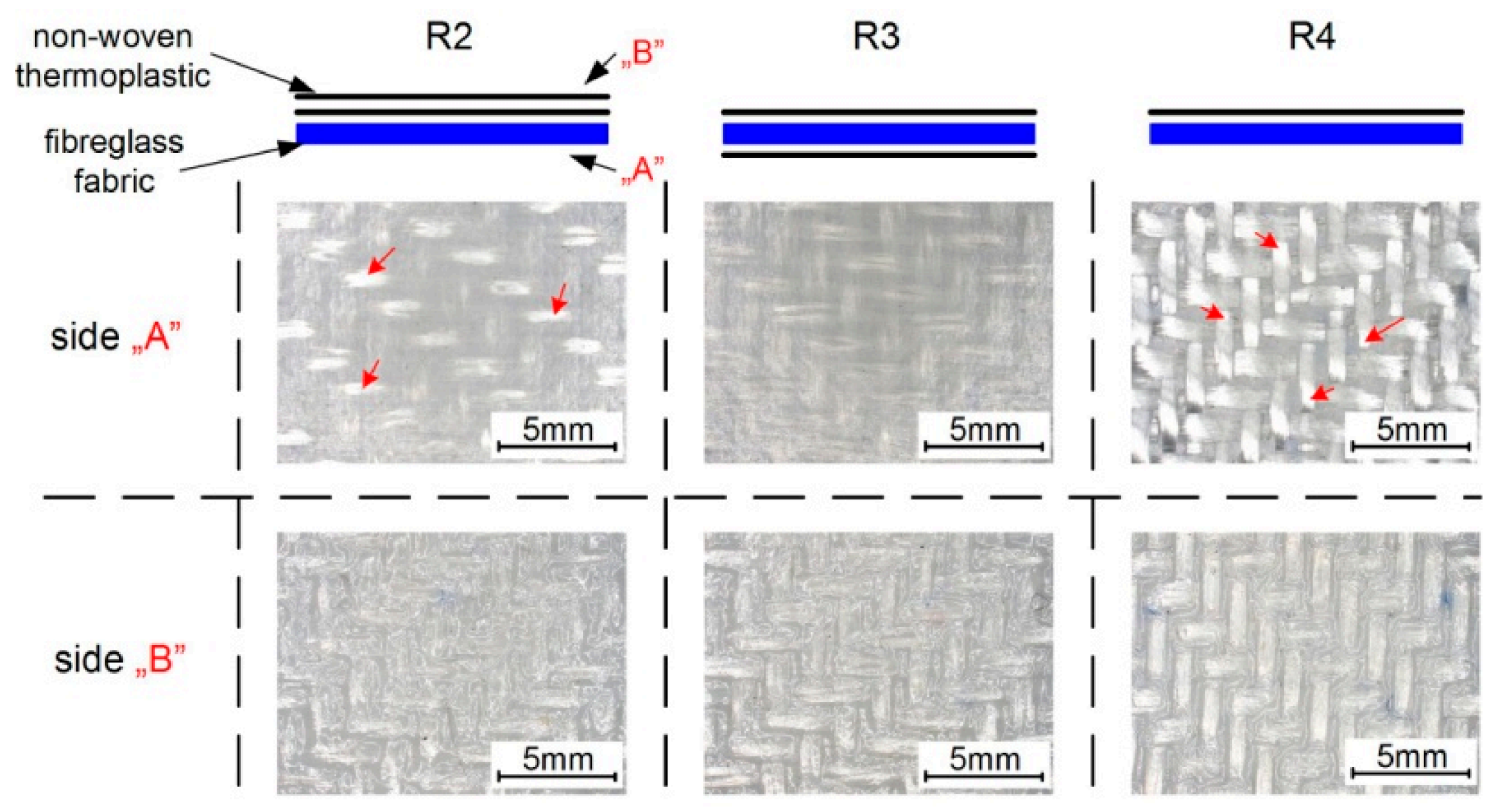
Microscope images of the surfaces of the reference specimens.

Reference specimens measuring 30 mm × 250 mm were prepared in an analogous manner, with the arrangement and number of thermoplastic nonwoven layers treated as the key variables (Figure 5). The R_1 specimens consisted solely of the glass fabric described above. The R_2 specimens contained two thermoplastic nonwoven layers, placed one on top of the other on the heated platen side of the press. Two thermoplastic nonwoven layers were also used in the R_3 specimens, arranged symmetrically: one on the press bed and the other on the heated platen side. The R_4 specimens contained only one thermoplastic nonwoven layer. This configuration was introduced as a simplified reference case representing a reduced amount of thermoplastic matrix available for glass-fabric impregnation. It was not intended to reproduce the detailed matrix-starved state occurring in an actual PC, where the powder grains additionally disturb thermoplastic flow and produce a spatially heterogeneous impregnation pattern. Figure 5 presents microscope images of the reference series, together with schematics of the layer arrangements prior to heat bonding.

The difference between batches R_2 and R_3 is clear: exposed fibres that were not impregnated by the matrix are visible in the R_2 specimens. A similar phenomenon may occur when a thin powder layer restricts matrix flow. Batch R_3 represents the ideal case, in which the entire fibre volume is impregnated, and no exposed tows are visible at the surface. In the R_4 specimens, the matrix volume was reduced by half, resulting in extensive areas of exposed fabric tows. It should be emphasised, however, that incomplete impregnation of the glass-fabric backing layer in the PCs was intentional. During processes such as vacuum infusion, the unimpregnated regions play an important role by forming a bond with the liquid resin. Image analysis of the surface micrographs in Figure 5 showed that the exposed-fibre surface fraction was lowest for R_3 (~1.5%), intermediate for R_2 (~5.4%) and highest for R_4 (~11.2%).

Accordingly, the R-series specimens should be regarded as reference configurations to isolate the effects of the amount and distribution of thermoplastic, rather than as exact physical replicas of the PC backing layer. In particular, R_3 represents a limiting case of nearly complete fabric impregnation, whereas R_4 provides a simplified low-matrix reference. The actual PC structure is more heterogeneous because the powder layer locally restricts thermoplastic flow and modifies the matrix distribution within the fabric.

Table 2 summarises the measured thicknesses and areal densities of the reference specimens (R series) and the pre-impregnated coatings (A series), together with the calculated constituent fractions. The glass-fabric backing layer had an areal density of 280 g/m^2^, whereas each thermoplastic nonwoven layer had an areal density of 79 g/m^2^. Therefore, the thermoplastic areal mass, m_TP_, was calculated as (Equation (2)):

(2) $$m_{TP}=n_{TP}\cdot m_{NW}$$

where *n_TP_* is the number of thermoplastic nonwoven layers and *m_NW_* = 79 g/m^2^ is the areal density of a single nonwoven layer.

For the A-series coatings, the retained powder areal mass, *m_P_*, was calculated by subtracting the known glass-fabric and thermoplastic contributions from the experimentally measured total PC areal density (Equation (3)):

(3) $$m_{P}=m_{PC}-m_{GF}-m_{TP}$$

where *m_PC_* is the measured total areal density of the PC and *m_GF_* = 280 g/m^2^ is the areal density of the glass-fabric backing layer.

The thermoplastic mass fraction was then calculated as (Equation (4)):

(4) $$w_{TP}=\frac{m_{TP}}{m_{PC}}\cdot 100\%$$

To provide an additional quantitative description of the powder loading, an apparent powder volume fraction was estimated from the retained powder mass, powder-material density and measured total PC thickness. It was defined as (Equation (5)):

(5) $$\phi_{P,app}=\frac{V_{P}}{V_{PC}}\cdot 100\%$$

where the powder volume is (Equation (6)):

(6) $$V_{P}=\frac{m_{P}}{\rho_{P}}$$

and *ρ_P_* is the density of the corresponding powder material. For a reference area of 1 m^2^ and a PC thickness t expressed in millimetres, the total geometrical PC volume is (Equation (7)):

(7) $$V_{PC}=1000\cdot t[{\mathrm{cm}}^{3}]$$

which gives the following convenient form (Equation (8)):

(8) $$\phi_{P,app}=\frac{m_{P}}{\rho_{P}\cdot 1000\cdot t}\cdot 100\%.$$

The calculated value should be interpreted as the apparent powder volume fraction within the overall geometrical volume of the complete PC. It does not represent the local packing fraction within the functional layer because the total PC volume also includes the glass-fabric backing layer, the thermoplastic matrix, and intergranular spaces.

The powder areal mass was calculated by subtracting the known contributions from glass fabric and thermoplastic from the measured total areal density. The apparent powder volume fraction was calculated using the powder and material densities reported in [33] and the measured total PC thickness. It therefore represents the powder fraction within the overall geometric volume of the PC, not the local packing fraction within the functional layer.

Although all A-series coatings contained the same thermoplastic areal mass of 158 g/m^2^, their thermoplastic mass fraction decreased from 24.6 wt.% for A_1 to 13.2 wt.% for A_5 because of the increasing retained powder mass. The calculated powder areal mass ranged from 204 g/m^2^ for A_1 to 760 g/m^2^ for A_5. The apparent powder volume fraction ranged from 8.5 to 22.1 vol.%. The stainless-steel-grit coating A_3 exhibited the lowest apparent powder volume fraction despite its relatively high powder areal mass, owing to the substantially higher density of the steel particles. These results demonstrate that powder mass, powder volume fraction, coating thickness and thermoplastic fraction varied simultaneously among the investigated A-series coatings. Consequently, the mechanical response of an individual batch should be interpreted as the response of the complete PC system rather than as the isolated effect of a single powder parameter.

The void fraction within the stand-alone PCs was not quantified in the present study. It should be noted that the spaces between powder grains in the as-manufactured PC do not necessarily represent conventional manufacturing defects, because partial open space within the granular layer is inherent to the proposed architecture and may subsequently be infiltrated by liquid resin during composite manufacture. Moreover, reliable two-dimensional quantification from conventionally prepared cross-sections is difficult for the stand-alone PC because the relatively compliant thermoplastic matrix does not rigidly retain the hard powder grains during cutting and polishing. Grain pull-out may therefore produce artificial cavities, leading to an overestimation of the actual void fraction. A non-destructive three-dimensional technique, such as X-ray micro-computed tomography, would be more appropriate for quantitatively assessing the internal void structure and will be considered in future work.

**Figure 6:**
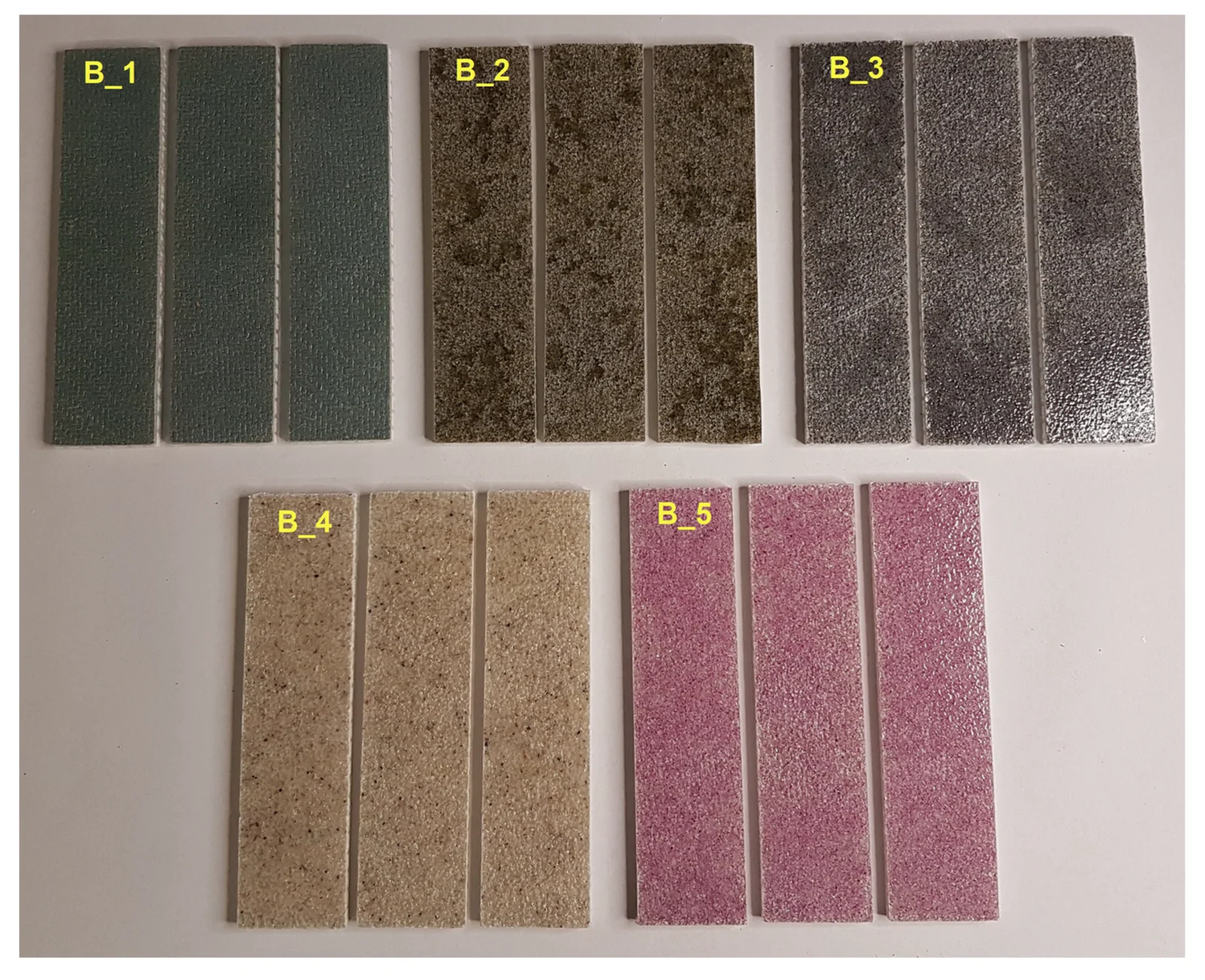
Specimens for the three-point bending tests.

**Figure 7:**
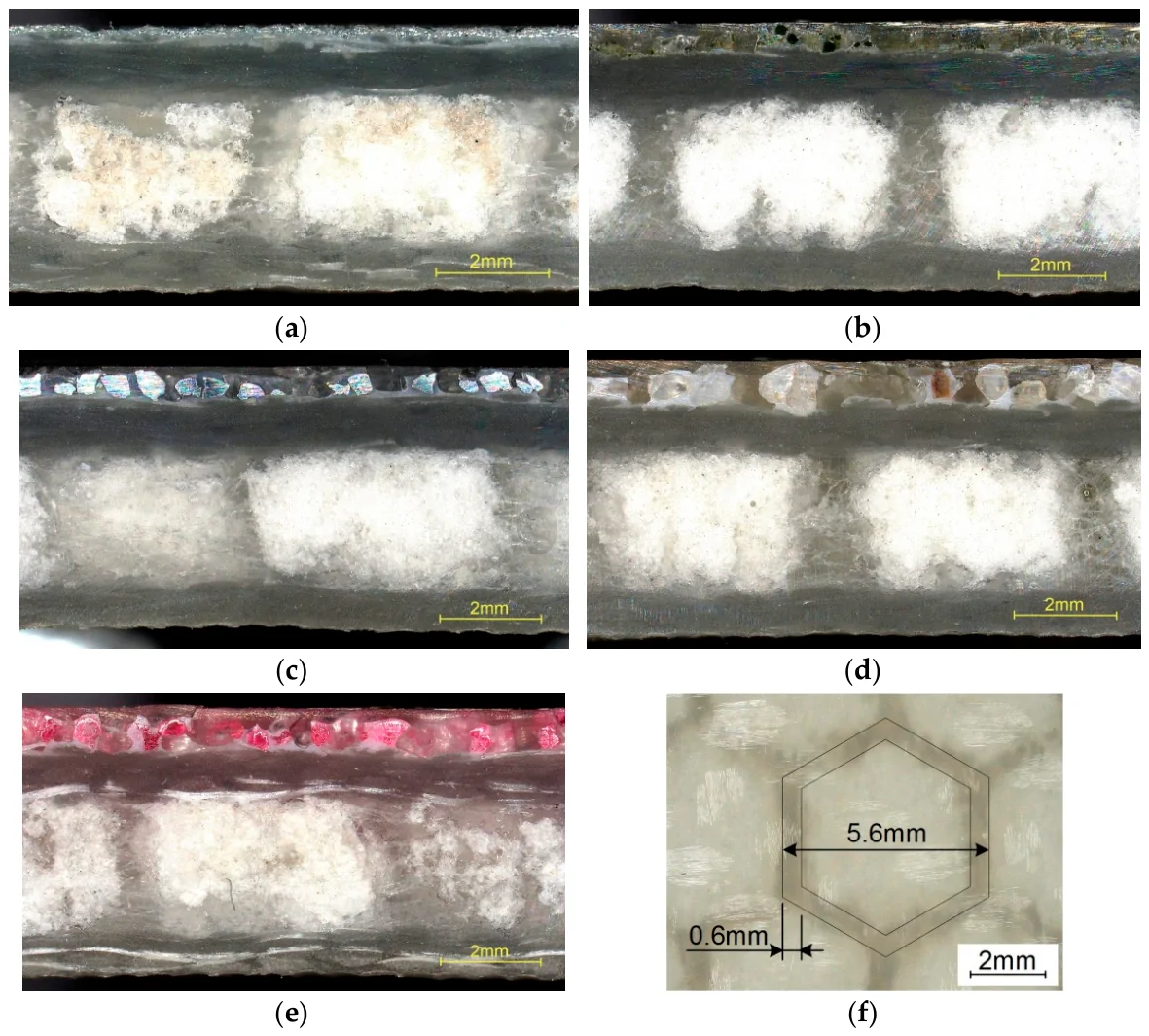
Cross-sections of sandwich composites with pre-impregnated coatings: (**a**) B1—green silicon carbide F220; (**b**) B2—glauconite F60; (**c**) B3—stainless-steel grit DELTA 050; (**d**) B4—quartz sand F80; (**e**) B5—chromium electrocorundum F36; (**f**) detailed view of the Soric SF core geometry.

The practical application of the PC concept was also demonstrated by manufacturing a sandwich composite using the vacuum-infusion procedure described in detail in [33]. Five 200 mm × 300 mm panels were produced, from which 30 mm × 120 mm batch-B specimens were cut for three-point bending tests. The specimens were machined with a 3 mm-diameter tool on a CNC router supplied by ATM Solutions (Łomianki, Poland). The prepared test specimens are shown in Figure 6. Figure 7 presents cross-sections of the investigated PC-integrated sandwich composites, in which the Soric SF core, laminate skins and PC layer can be distinguished; the PC thickness differed between batches. No PC delamination was observed. The backing layer merged smoothly into the laminate, and the interface could not be clearly distinguished, indicating effective integration of the coating system. No macroscopic impregnation defects or interfacial discontinuities were observed in the examined cross-sections. However, the degree of infiltration of the PC backing layer by the infusion resin was not quantified by image segmentation. Such an analysis would require multiple representative polished cross-sections from each PC variant and a dedicated segmentation procedure capable of distinguishing the thermoplastic matrix from the cured infusion resin. The microscopy performed in the present study was intended primarily to verify the structural integration of the PC with the sandwich laminate rather than to determine local resin volume fractions. The observations were performed using a Keyence VHX-970F digital microscope (Osaka, Japan). Measurement of the Soric SF core geometry, shown in Figure 7f, was important for the numerical modelling.

Because all sandwich panels were manufactured using the same laminate lay-up, consisting of three glass-fabric plies on each side of the Soric SF core, the nominal GFRP skin architecture was identical for all B-series specimens. However, the thickness of the integrated PC varied between batches, thereby affecting the total sandwich thickness. To distinguish this geometrical effect from differences associated with the PC material system, the actual post-infusion thicknesses were measured from the cross-sections shown in Figure 7 and are summarised in Table 3.

The layer thicknesses were determined at ten evenly distributed locations across each microscope cross-section and are reported as mean ± standard deviation. The standard deviation therefore represents local thickness variation within the examined cross-section. The principal geometrical difference was associated with the PC layer, whose thickness varied between batches. Consequently, the differences observed in the bending response may reflect both the properties of the individual PC systems and the resulting variation in cross-sectional geometry.

**Figure 8:**
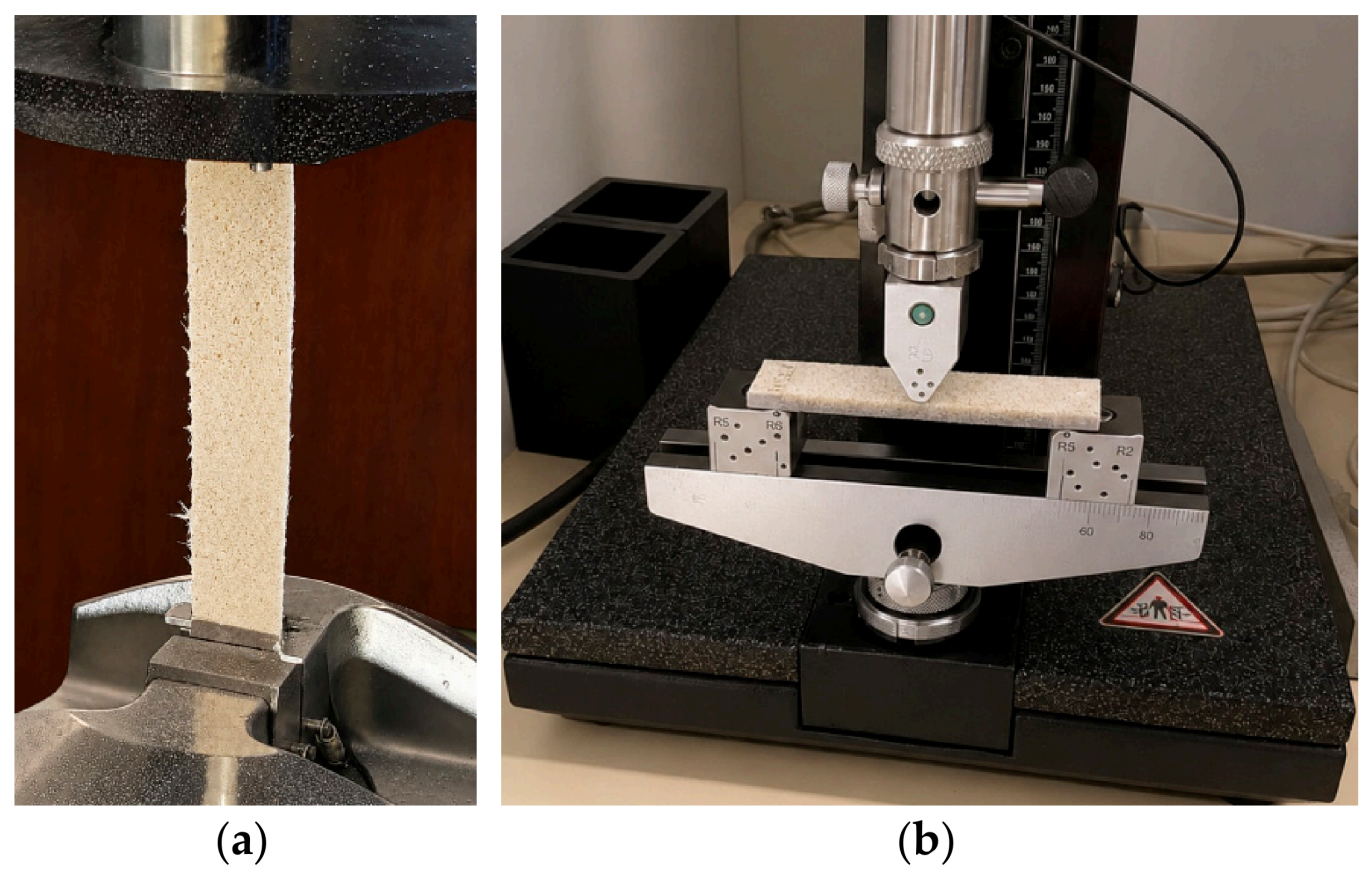
Laboratory tests: (**a**) uniaxial tensile testing of the pre-impregnated coatings; (**b**) bending of the sandwich specimens.

After preparation, the reference and PC specimens were subjected to uniaxial tensile testing (Figure 8a) using an MTS 100 kN universal testing machine (MTS Systems Corporation, Eden Prairie, MN, USA). To improve load transfer and prevent local damage in the gripping regions, 30 mm × 50 mm end tabs cut from 1 mm-thick PF CC 201 laminate (Izo-Erg, Gliwice, Poland) were bonded to both ends of each specimen. The resulting gauge length between the tabs was 150 mm. The tests were performed at a crosshead displacement rate of 1 mm/min, with three specimens tested for each batch. An ARAMIS DIC system (Carl Zeiss, Oberkochen, Germany) was used to monitor the strain field over a 30 mm × 150 mm measurement area.

Because the specimens exhibited different degrees of initial curvature and progressive straightening, a single fixed strain interval was not imposed. For each specimen, the apparent Young’s modulus and apparent Poisson’s ratio were determined from the same approximately linear portion of the DIC response, selected after the initial nonlinear stage. The lower limits of the selected longitudinal-strain intervals ranged from approximately 0.57 to 1.19%, whereas the upper limits ranged from approximately 1.20 to 1.85%.

The three-point bending tests were performed using a Zwick/Roell testing machine equipped with a 2.5 kN load cell (ZwickRoell GmbH & Co. KG, Ulm, Germany) (Figure 8b). The support span was 100 mm, and the tests were conducted at a crosshead displacement rate of 5 mm/min. Three specimens were tested for each B-series batch.

## 3. Representative Models of the PC and PC-Integrated Composite

Representative models of both the PC and the PC-integrated sandwich composite were developed in order to:

- Demonstrate the capabilities of the original algorithm for generating grain layers;
- Assess the feasibility of integrating relatively large assemblies with contact pairs into an FE environment;
- Determine the effect of powder-layer grit size on the mechanical response of the constituent materials.

At this stage, the numerical framework was intentionally restricted to the pre-damage mechanical response. The principal objective was to establish the computational feasibility of a geometrically detailed model that explicitly represents the fabric, thermoplastic matrix, and individual powder grains, and subsequently to compare the relative responses of models with different grit sizes under identical loading conditions. Material damage evolution, stiffness degradation, interfacial debonding and progressive failure were therefore not included. Such an approach is consistent with micromechanical FE studies in which linear-elastic representative models are used to investigate effective behaviour and local stress and strain distributions before more complex failure mechanisms are introduced [35,36].

**Figure A1:**
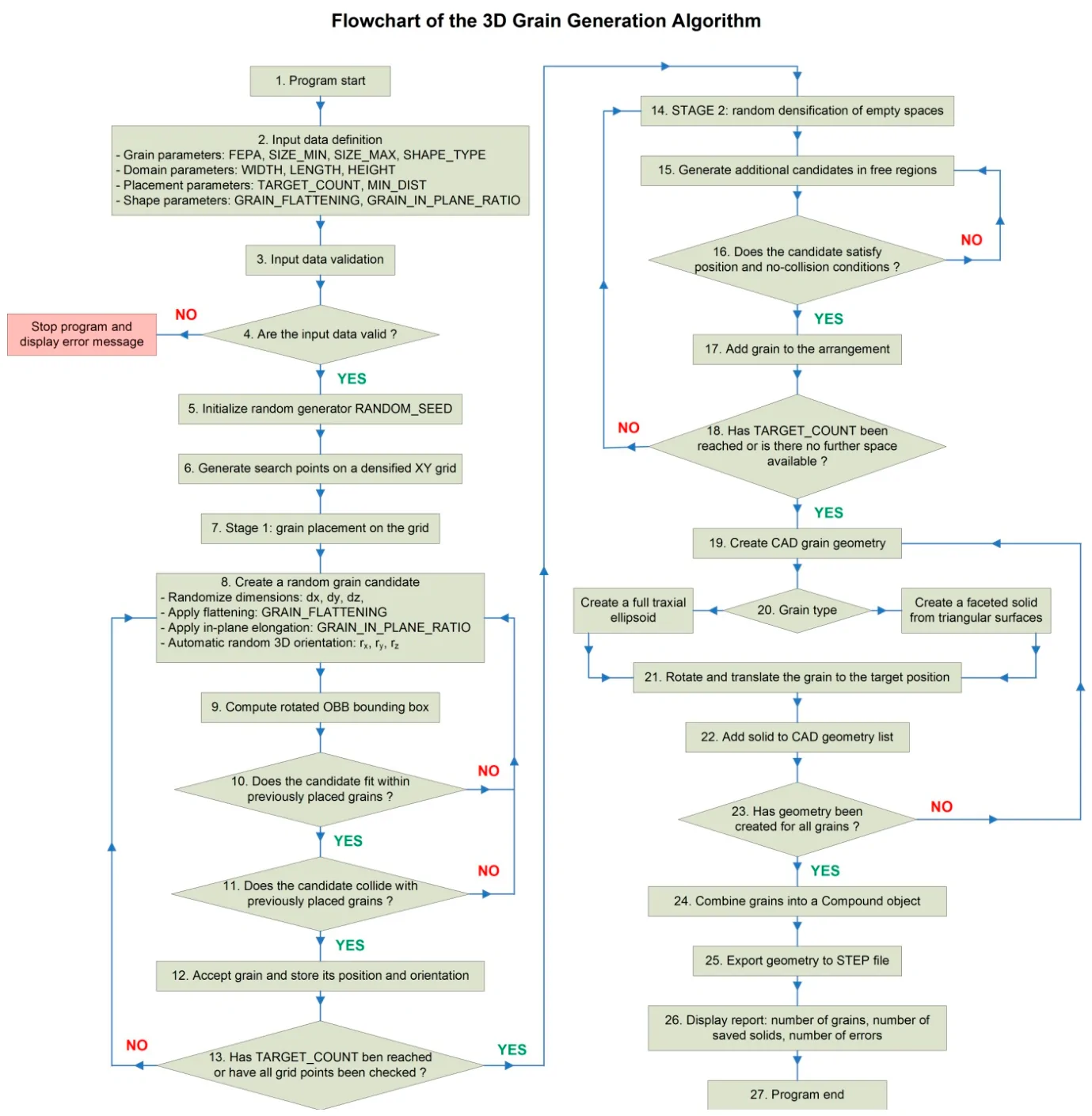
Flowchart of the 3D grain-generation algorithm.

The common features of both RVE types were a powder layer embedded in a thermoplastic matrix and overall in-plane dimensions of 10 mm × 20 mm, and were selected to provide a sufficiently large population of randomly distributed grains while maintaining a computationally manageable CAD and FE model. The ellipsoidal F24, F30 and F36 models contained 205, 283 and 394 grains, respectively. These populations are comparable to or larger than those commonly used in particle-based representative volume models. Cugnoni and Galli [37] showed that the characteristic RVE dimension of random particle-reinforced composites was approximately 5–6 times the particle size at reinforcement volume fractions of 5–10% and increased to approximately 10–20 times the particle size at 15–25%. Other numerical studies employed RVEs containing 32–147 randomly distributed particles in particle-reinforced polymer composites [38] and 50 particles in numerical models of compacted powders [39]. Using the characteristic FEPA grain dimensions, the shorter 10 mm side of the present models corresponds to approximately 13–19 characteristic grain sizes for F24–F36, while the 20 mm dimension corresponds to approximately 27–38 grain sizes. The selected domain was therefore considered sufficiently large for the comparative analysis of local stress, strain and displacement fields performed in this study. A purpose-built Python 3.12 algorithm was developed; its flowchart is provided in Appendix A (Figure A1).

The principal tool used by the algorithm is CadQuery 2.8.0, a library for scripted, parametric creation of three-dimensional CAD (computer-aided design) models. Its capabilities include surface and solid modelling, CAD data exchange and support for neutral formats such as STEP (Standard for the Exchange of Product Model Data) and IGES (Initial Graphics Exchange Specification). From an engineering perspective, CadQuery is not merely a STEP converter but a geometry-creation environment. It enables models to be built using concise scripts, dimensions to be defined as parameters, and standard CAD operations, including extrusion, revolution, filleting, chamfering, Boolean operations, solid partitioning, and spatial transformations. Crucially, it also supports the creation of assemblies.

In the literature, CadQuery is presented primarily as a tool for scripted, parametric CAD modelling rather than simply a library for exporting geometry to STEP. Machado et al. [40] analysed open-source tools for designing scientific equipment and identified CadQuery, a Python library that enables the creation of parametric models with relatively concise code. They also highlighted its ability to export models in standard parametric formats, which is important for subsequent processing in CAD/CAE systems. More recent studies have used CadQuery as part of design-automation workflows. Schöfer and Seibel [41] employed it to support parametric design processes using large language models, treating CadQuery code as an intermediate, editable representation of geometry. A similar direction is evident in work on generating CAD models from textual descriptions. Xie and Ju [42] proposed a Text-to-CadQuery approach in which language models generate CadQuery code directly, avoiding less transparent task-specific CAD representations. Guan et al. [43], in turn, presented CAD-Coder, in which CadQuery serves as a parametric CAD language that supports both model generation and geometric validation. Collectively, these studies show that CadQuery can be used as a parametric tool for both practical and scientific purposes, automating and accelerating preparation of models for FE simulation.

The algorithm shown in Figure A1 begins by defining the principal input parameters: FEPA grit designation, grain-size range, dimensions of the placement area, target number of grains and minimum distance between neighbouring grains.

In the first stage, the input data are validated, and a random-number generator is initialised, allowing the same grain arrangement to be reproduced for a given seed value. A dense grid of points is then created in the XY plane. These points serve as potential grain locations during the first placement stage. For each grid point, the programme generates a set of random grain candidates and assigns a random three-dimensional orientation. Collisions are checked using bounding boxes to prevent grain interpenetration. This approach, however, leaves a relatively large amount of unused space between grains, and the algorithm was therefore divided into two stages.

After grid-based placement, the second stage performs random densification of the remaining voids. The programme generates further random grains and attempts to place them in available spaces until either the target grain count is reached or further densification becomes ineffective. The actual CAD geometry is then created. For the ‘smooth’ grains, a complete triaxial ellipsoid is generated with dimensions corresponding to the previously sampled values of dx, dy and dz. For the ‘sharp’ grains, a faceted solid is generated to provide a more realistic representation of electrocorundum and other materials with angular edges.

**Figure 9:**
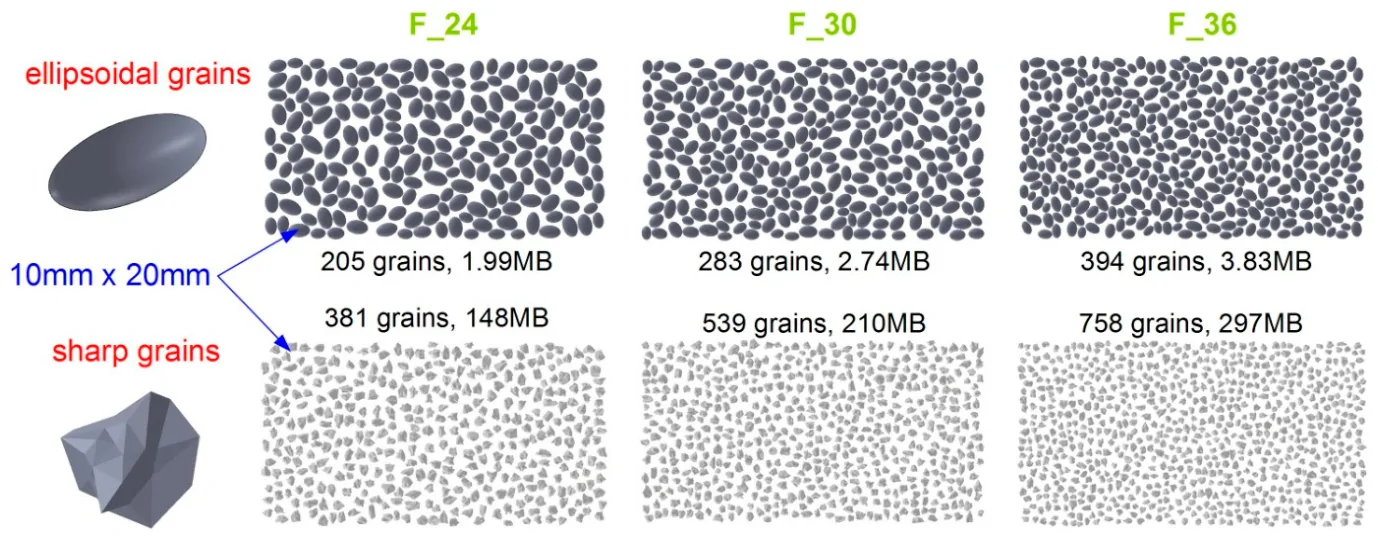
Powder layers generated by the algorithm.

Finally, the geometry is exported to a STEP file, along with the number of generated grains. Figure 9 shows the ellipsoidal and sharp-edged grains generated for the F24, F30 and F36 grit sizes. For a given grit size and surface area, considerably more sharp-edged grains than smooth grains can be placed. This is because the ellipsoidal grains are elongated; however, elongation does not affect sieve classification, which is determined by the shorter axis. Only ellipsoidal grains, whose shapes are similar to those of sand grains, were used in the subsequent model preparation. STEP file size also influenced this choice, as indicated for each grit size in Figure 9. Files containing smooth grains were several times smaller. Because this modelling approach was being tested for the first time, the more robust option was selected.

The repeatability of the stochastic CadQuery grain-generation procedure was additionally evaluated using three independent geometrical realisations for each grit-size case (F24, F30 and F36), generated with different random seeds. For this verification, the adaptive filling stage was disabled to reduce computational cost, while all other generation parameters were kept unchanged. The resulting grain counts, total grain volumes and grain surface areas are summarised in Table 4.

**Figure 10:**
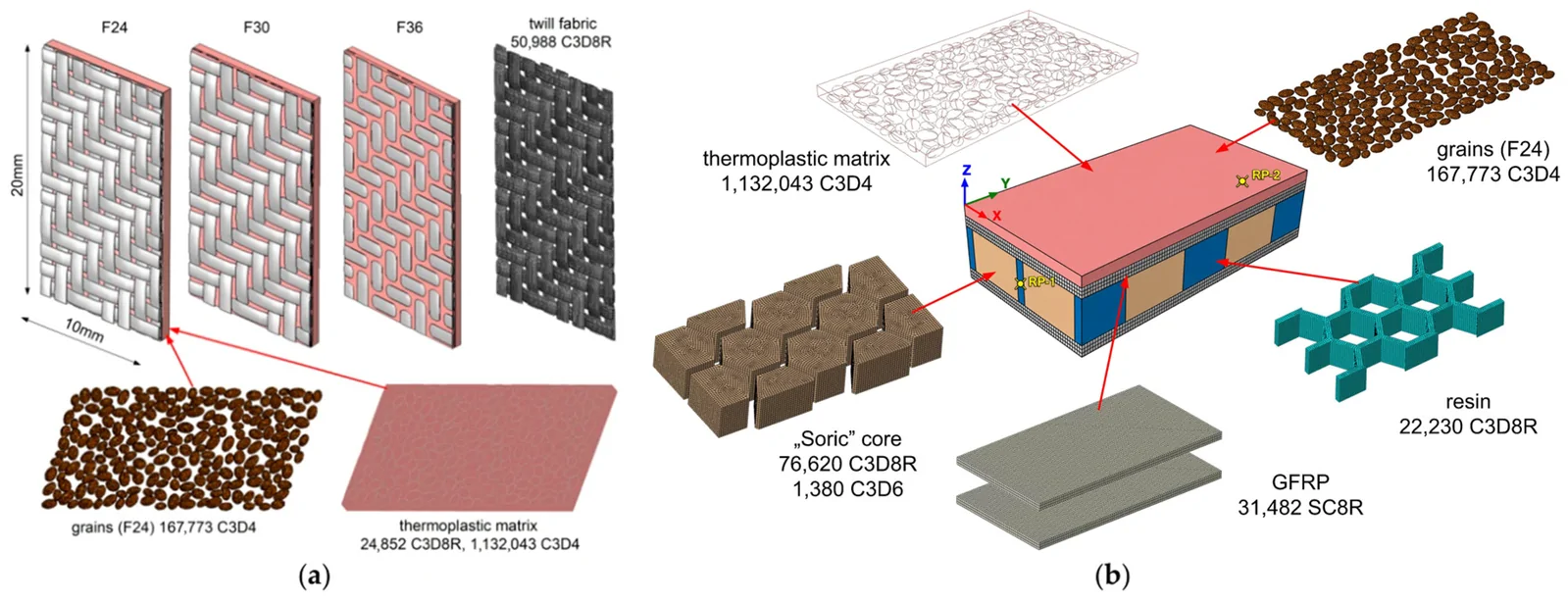
Preparation of the representative FE models: (**a**) pre-impregnated coating models; (**b**) PC-integrated sandwich-composite model.

Figure 10 presents two types of RVE: Figure 10a the PC model and Figure 10b the PC-integrated sandwich-composite model. For each type, three cases with different grain sizes, F24, F30 and F36, were considered. The numerical models were not intended to reproduce the individual powder materials used in the experimental programme. Instead, they were formulated as a controlled parametric study of grain size. Therefore, identical elastic properties were assigned to the grains in all numerical variants, while the grain dimensions and resulting grain population were varied. This approach eliminates the influence of material stiffness on the comparison and allows the effect of grain size on load transfer and on local stress, strain, and displacement fields to be assessed independently. Accordingly, the F24, F30 and F36 numerical variants should be interpreted as generic geometric grit-size models rather than numerical representations of the specific silicon carbide, glauconite, stainless steel, quartz or chromium-electrocorundum coatings investigated experimentally.

Using different grain sizes with the same amount of thermoplastic matrix in the coating results in varying degrees of fibre impregnation in the backing fabric. Filling the void space between coarser grains requires considerably more matrix than filling the spaces between finer grains. Consequently, for the finer grit designation, a larger proportion of the glass-fabric fibres can be impregnated than for the coarser grade. This effect is clearly illustrated by the difference between specimens R_3 and R_4 in Figure 5, where the matrix content of R_4 was reduced by half. In PCs, some fibres that remain unimpregnated by the thermoplastic matrix are desirable because they can bond to the liquid resin during processes such as vacuum infusion. Two limiting cases may nevertheless occur:

- The thermoplastic matrix may have only minimal contact with the backing layer;
- The thermoplastic matrix may completely impregnate the backing-layer fibres.

In the first case, the coating may readily delaminate from the backing layer after the PC-integrated composite has been manufactured. In the second, a durable bond between the PC and the composite may not form because no unimpregnated fibres remain to bond with the liquid resin. Cross-sectional microscopy and microscale numerical modelling are therefore essential. The models shown in Figure 10a used the same volume of thermoplastic matrix; the matrix solid was obtained by subtracting the grain models and part of the fabric-tow volume. The same thermoplastic-matrix volume was deliberately used in all grit-size models. This assumption was consistent with the laboratory manufacturing procedure, in which the thermoplastic content was controlled by the number of nonwoven layers with a fixed areal density (79 g/m^2^) rather than being adjusted continuously. Consequently, the matrix amount could only be varied in discrete increments by adding or removing complete nonwoven layers. The reference specimens demonstrated that even such a one-layer change strongly affected fabric impregnation. Increasing the thermoplastic content further would also progressively fill the spaces between the grains and encapsulate a larger fraction of their surfaces, thereby reducing the distinct granular character of the functional layer. From a numerical perspective, keeping the matrix volume constant was also necessary to isolate the effect of grit size: varying both grain dimensions and matrix content simultaneously would prevent the observed differences in stress, strain, and displacement fields from being attributed to grit size. The adopted models should therefore be regarded as a controlled parametric study rather than as an attempt to reproduce an independently optimised matrix volume for each grit grade.

**Figure 12:**
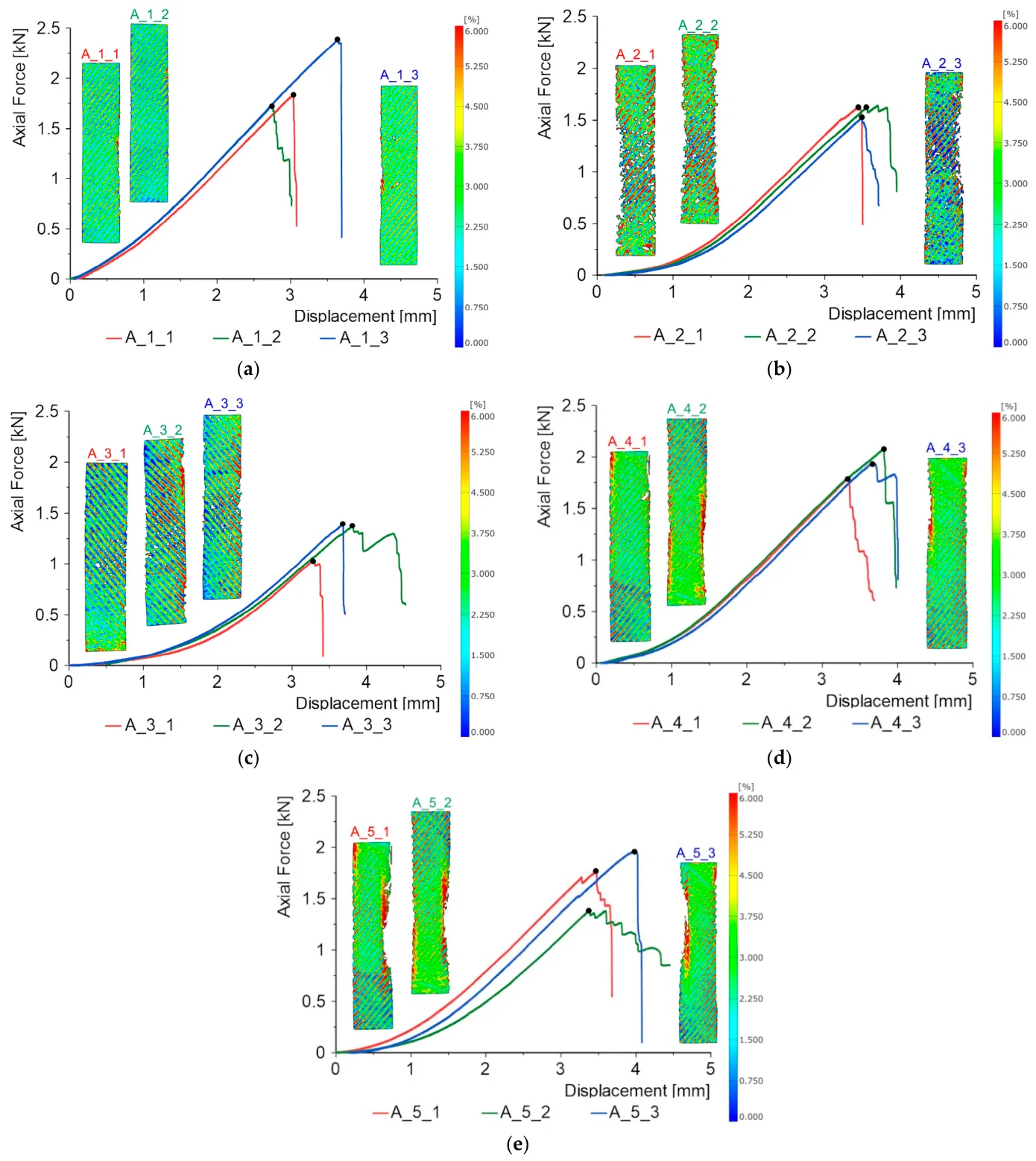
Force–displacement curves and strain maps for the pre-impregnated coatings: (**a**) batch A1—green silicon carbide; (**b**) batch A2—glauconite; (**c**) batch A3—stainless steel grit; (**d**) batch A4—quartz sand; (**e**) batch A5—chromium electrocorundum.

The CAD models were prepared in SolidWorks 2014 (Dassault Systèmes, Waltham, MA, USA). The fabric model reproduced the laboratory geometry, using a 280 g/m^2^ twill weave. A sweep operation was used, and the surface of each tow in the CAD model was divided into two halves to avoid later Abaqus meshing problems and to facilitate the use of more regular C3D8R elements. C3D4 elements were used primarily for the grains and matrix because they conform more readily to irregular surfaces. The analyses were conducted in Abaqus/Standard (Dassault Systèmes, Vélizy-Villacoublay, France). Tie constraints were applied between contacting pairs. The PC models were then subjected to uniaxial tension along the longer edge using a prescribed displacement of 0.15 mm. This corresponded to a displacement of 3.38 mm in the global model, assuming a 30 mm × 150 mm gauge section matching the actual specimen, after the gripped regions were excluded. According to the curves in Figure 12, this displacement corresponds to the region of maximum force.

The RVE shown in Figure 10b was subjected to a bending moment of 6.67 Nm, corresponding to a bending force of 800 N, assuming that the fragment was extracted from the central region of the actual specimen. This load was below the experimental maximum force of approximately 930–960 N and was selected to compare the numerical variants in the pre-peak regime. Damage evolution was not defined for any material because the objective of the model was to compare stress, strain and displacement fields prior to global failure rather than to reproduce the complete experimental failure process.

As in the PC simulations, tie constraints were applied between all contact pairs. This assumption was intended to represent the fully bonded condition of the constituents after manufacture. In the PC-integrated sandwich specimens, regions of the glass-fabric backing layer that were not impregnated by the thermoplastic matrix during heat pressing were subsequently infiltrated by the polyester resin during vacuum infusion. After curing, these regions therefore formed a continuous bonded connection with the surrounding laminate. Cross-sectional microscopy supported this assumption, as no macroscopic PC delamination was observed and the backing layer merged smoothly with the laminate. Nevertheless, the tie formulation represents an idealised, perfectly bonded interface and cannot reproduce local interfacial compliance, slip, debonding or progressive separation. It may therefore influence the predicted local stress transfer, particularly near material interfaces. For this reason, the present numerical results are interpreted primarily in terms of comparative pre-damage stress, strain and displacement fields.

The material data used in both types of simulation are summarised in Table 5 and were based on literature data and the authors’ own test results.

## 4. Results and Discussion

This section presents and discusses the results of uniaxial tensile tests on the reference and PC specimens, as well as three-point bending tests on PC-integrated sandwich composites. The experimental analysis was supplemented by DIC results and FE simulations of representative structural models. Particular attention was paid to differences among the complete PC systems, the layered architecture and the degree of fabric impregnation.

### 4.1. Uniaxial Tensile Tests

**Figure 11:**
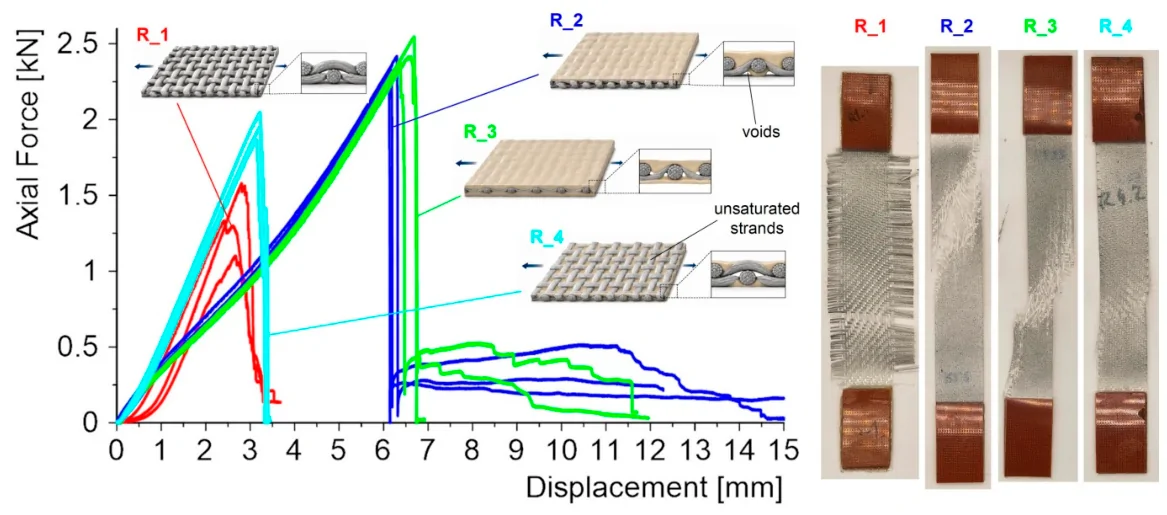
Force–displacement curves for the reference specimens.

The reference specimens formed an important material group because their results provided a basis for comparison with the PC specimens later in the discussion. The force–displacement curves for the reference specimens, shown in Figure 11, also demonstrate how the mechanical response changes with the arrangement and number of thermoplastic nonwoven layers.

The R_1 specimens, made solely from glass fabric, exhibited the lowest failure force. Examination of all the curves, however, shows that not only the maximum values but also the curve shape and repeatability are important. Batch R_1 displayed poor repeatability in both curve shape and maximum force. This was caused by the greater difficulty of preparing these specimens, particularly in maintaining the same number of weft tows and hence a consistent width. A further challenge was bonding the end tabs, which had to transfer the load uniformly from the grips to the individual fibre tows. Non-uniform resin impregnation or adhesive layer discontinuities resulted in uneven loading of the specimen. As shown in Figure 11, the reference specimens failed across a section perpendicular to the tensile axis.

A comparison of batches R_2 and R_3 is particularly informative. Their maximum forces were 176% and 182% higher, respectively, than those of batch R_1. In principle, R_2 and R_3 should have exhibited the same properties because they contained the same amount of matrix. In this case, however, the arrangement of the thermoplastic nonwoven layers, shown in Figure 5, was decisive. Microscopy revealed exposed-fibre regions in the R_2 specimens. This is reflected in Figure 11, where the failure force of R_2 was approximately 3.7% lower than that of R_3. Both R_2 and R_3 failed along planes inclined at approximately 45°, corresponding to the planes of maximum shear stress.

The final reference group, R_4, contained half as much thermoplastic matrix as the two preceding batches. This reduced the maximum force by approximately 20.4% relative to R_3, although the value remained 45.5% higher than that of R_1. The failure mode also changed, with the fracture no longer occurring perpendicular to the tensile axis.

Figure 12 presents the force–displacement curves for the PC specimens, which contained two thermoplastic nonwoven layers arranged as in the R_2 specimens, in addition to the glass fabric and powder layer. In every case, introducing the powder layer reduced the maximum force by between 16.1% (batch A_1) and 46.6% (batch A_3). This reduction was associated with the smaller amount of matrix present in the backing layer. The A-series specimens exhibited different force–displacement curve shapes; however, these differences cannot be attributed solely to the powder material, as powder type, grit size, and other coating characteristics varied simultaneously across batches. Two response groups can be distinguished: batches A_2–A_5 and A_1. The first group showed pronounced initial curvature up to approximately 1.5–2 mm of displacement, after which a linear segment began. The response of A_1 alone remained approximately linear throughout the test. A complete explanation of this phenomenon will require further tests on different grit sizes of the same material, combined with microscopic examination of cross-sections. Failure evolution was either abrupt, with the force falling to zero, as for specimen A_1_3, or gradual, with the force increasing slightly again after the maximum and the initial drop, as for specimen A_3_2. Consequently, the experimental results do not permit the independent effects of powder material and grit size to be decoupled. Controlled experiments using the same powder material at several grit grades would be required for such an assessment. The numerical study should therefore be regarded separately as a controlled parametric investigation of grain size, in which the grain material and thermoplastic-matrix volume were kept constant.

The DIC maps in Figure 12 are shown at the maximum force, immediately before the abrupt load drop. They reveal heterogeneous strain fields with bands aligned with the tow directions of the twill fabric. Higher strains occurred locally near specimen edges and in regions where the continuity of the powder or thermoplastic layer was disrupted. This indicates that PC failure did not result solely from a single constituent exceeding its strength, but from the interaction among the fabric deformation, the thermoplastic matrix, and the discontinuous powder layer.

**Figure 13:**
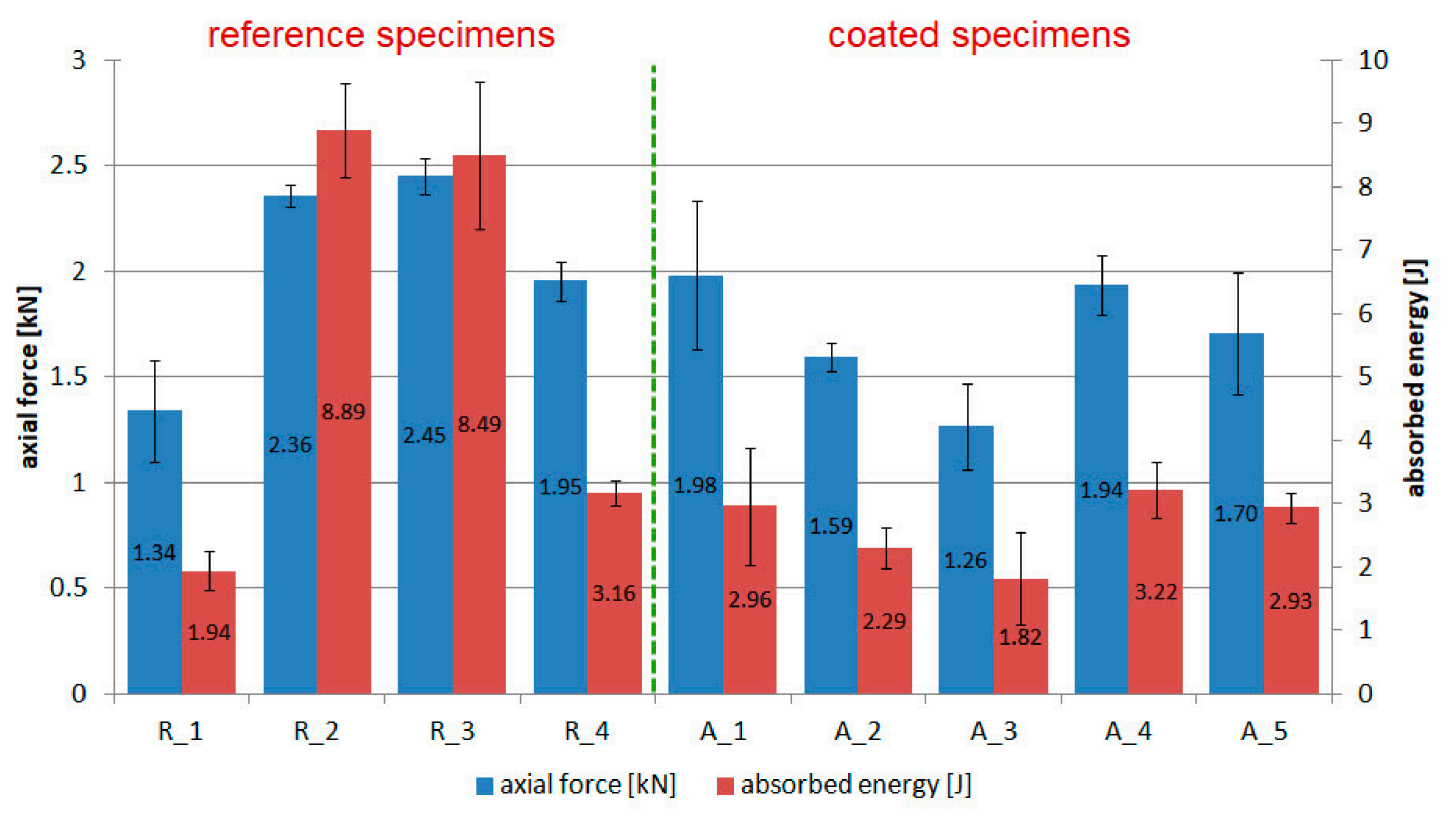
Tensile-test results for the reference specimens and pre-impregnated coatings.

Figure 13 provides a detailed comparison of the maximum forces and absorbed energies. For all specimens, the absorbed energy was calculated by numerically integrating the force–displacement curve from the start of loading to the end of the test, using the same integration criterion. As with the maximum force, the highest absorbed energies were obtained for batches R_2 and R_3. For the PC specimens, the absorbed energy was lower than that of batch A_1 by factors ranging from 2.76 (batch A_4) to 4.88 (batch A_3). In terms of percentage relative standard deviation (%RSD), batch A_2 gave the best results among the PCs: 4.02% for maximum force and 13.86% for absorbed energy.

Analysis of Figure 12 and Figure 13 does not reveal a simple relationship between the areal density or thickness of a PC and its maximum force. Several parameters changed simultaneously: powder material, density and grain arrangement, functional-layer thickness, and degree of glass-fabric impregnation. For example, batches A_1 and A_4 reached similar maximum forces despite clear differences in thickness and areal density. The results for each batch should therefore be regarded as characteristics of the complete material system rather than as direct measures of the effect of any single powder property.

**Figure 14:**
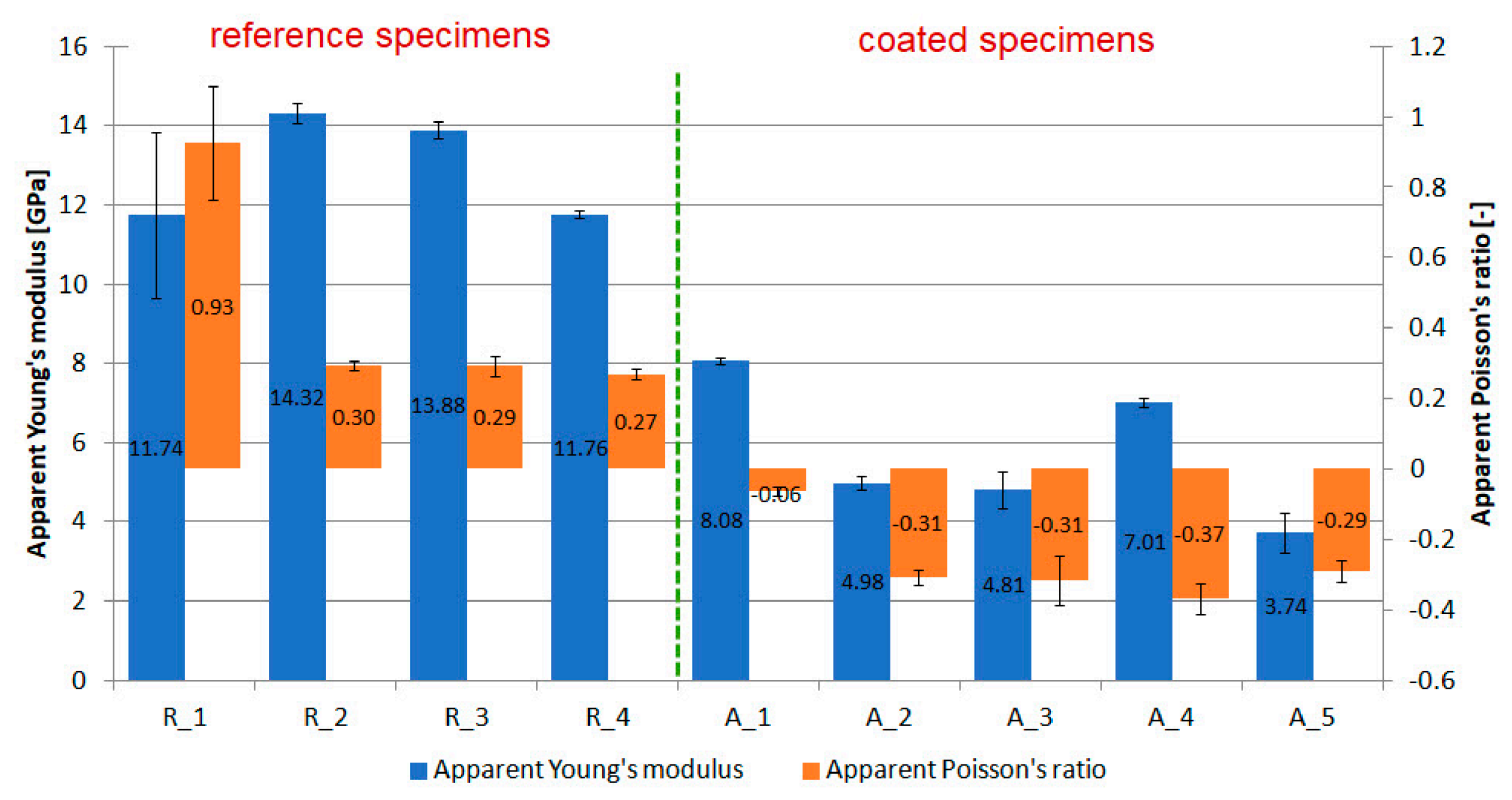
Apparent Young’s modulus and apparent Poisson’s ratio.

Figure 14 compares the apparent Young’s modulus and apparent Poisson’s ratio. These values should be interpreted as effective properties of the entire layered structure rather than as conventional intrinsic material constants. Stress was calculated using the total specimen thickness, including the grains and intergranular spaces. Increasing the thickness of the powder layer, therefore, increased the assumed cross-sectional area without necessarily producing a proportional increase in force, owing to the lower degree of glass-fabric impregnation. The apparent Young’s modulus of the reference specimens ranged from 11.74 to 14.32 GPa. For the PCs, it ranged from 3.74 GPa for series A_5 to 8.08 GPa for series A_1. All A-series variants, therefore, exhibited a lower apparent modulus than the reference specimens.

The results presented in Figure 14 require particular attention due to the high Poisson’s ratio measured for the glass fabric and the negative values obtained for the PC specimens.

For specimen R_1, a value greater than 0.5 should not be interpreted as violating the constraints of classical elasticity. The upper limit of 0.5 applies to a homogeneous, isotropic, linearly elastic continuum. A free fabric is instead an open geometrical structure whose deformation occurs primarily through changes in tow crimp, position and mutual contact between the warp and weft. During tension, the tows aligned with the loading direction straighten, and their curvature decreases. At the same time, the transverse tows become more crimped, move relative to one another and may be drawn towards the specimen axis. This mechanism can produce a transverse strain comparable to, or even greater than, the longitudinal strain. Sun, Pan and Postle showed that the Poisson effect in fabric arises from mechanical and geometrical interactions between the warp and weft, and that its magnitude depends on yarn stiffness, crimp and weave-structure parameters [48]. Penava et al. experimentally obtained maximum values of 1.39 and 1.18 for cotton fabric loaded in the two principal directions, and 0.88 and 0.68 for wool fabric [49]. The value of 0.93 obtained in the present study is therefore high but remains within the range reported for unimpregnated woven structures.

After the thermoplastic nonwoven was added, the apparent Poisson’s ratio of batches R_2–R_4 ranged from 0.27 to 0.30. The continuous matrix stiffened the interlacing regions, restricted changes in the tow crimp and reduced transverse tow movement. The response of these specimens was therefore closer to that of a thin laminated composite than to that of a free fabric.

**Figure 15:**
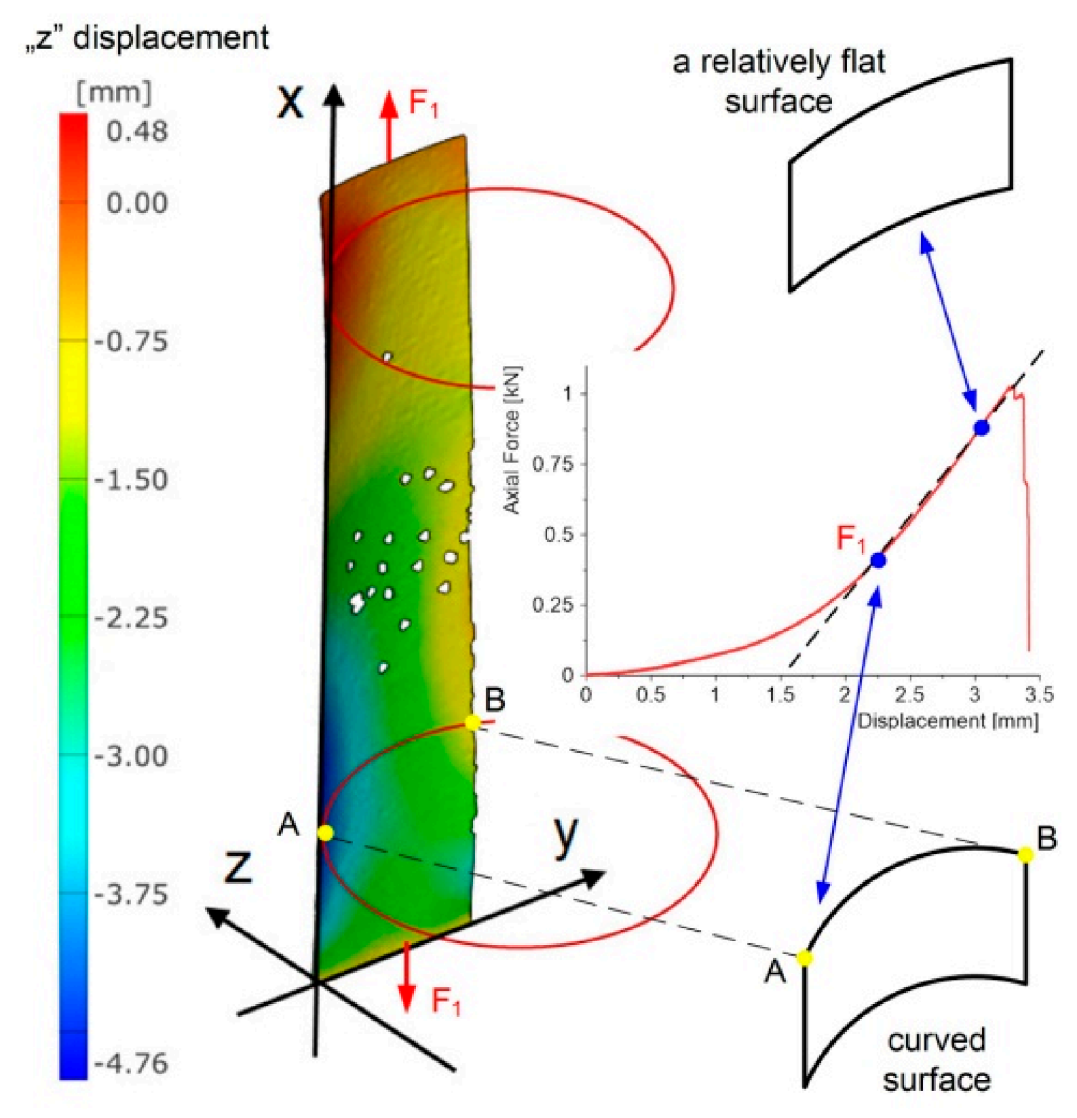
Schematic response of the pre-impregnated coating during tensile loading.

All A-series specimens exhibited negative apparent Poisson’s ratios, ranging from approximately −0.06 to −0.37. This does not mean that the coating constituents are auxetic materials or that the negative Poisson’s ratio is an intrinsic material constant. The effect results from the specimen curvature generated during heat bonding, visible, for example, in Figure 4. This curvature arises from both the asymmetry of the PC lay-up and differences in the thermal expansion of the constituent materials. During tension, the curved specimen was progressively straightened, as shown in the actual DIC image in Figure 15.

To quantify the initial out-of-plane deformation of the tensile specimens, the three-dimensional surfaces recorded by the optical system were exported as STL files and analysed using transverse Y-Z sections. Sections were extracted at 20 mm intervals along the specimen length, excluding the end regions. For each section, an equivalent circular radius was determined from the transverse profile. Because the radius varied along the specimen length, the values obtained from all valid sections were averaged for each specimen. The mean values for the three specimens in each series are reported in Table 6.

The analysis of the initial three-dimensional geometry confirmed that all tensile specimens exhibited measurable out-of-plane deformation. The smallest mean equivalent radii were obtained for the stainless-steel-grit A_3 and quartz-sand A_4 specimens, at 38.6 ± 0.4 mm and 41.0 ± 0.6 mm, respectively, indicating the most pronounced initial curvature. Intermediate values were obtained for the green-silicon-carbide A_1 specimens (54.8 ± 2.6 mm) and glauconite A_2 specimens (62.5 ± 3.3 mm), whereas the chromium-electrocorundum A_5 specimens exhibited the largest mean equivalent radius (130.6 ± 27.7 mm). Thus, the initial specimen geometry was not perfectly flat, and the magnitude of the out-of-plane deformation differed between the investigated series. These results support the interpretation that the apparent transverse expansion observed during tensile loading was influenced by progressive straightening of the initially curved specimens rather than representing an intrinsic negative Poisson’s ratio of the material, which is defined by Equation (9).

(9) $$\nu =-\frac{\varepsilon_{y}}{\varepsilon_{x}}$$

The literature shows that a negative Poisson’s ratio in woven structures can result from deliberately designed weave geometry, rotation of structural elements or differential shrinkage of individual regions [50]. No intentionally auxetic geometry was used in the specimens analysed here. It is therefore more appropriate to attribute the measured response to the straightening of the asymmetric coating than to classify the developed material as auxetic.

### 4.2. Three-Point Bending Tests

**Figure 16:**
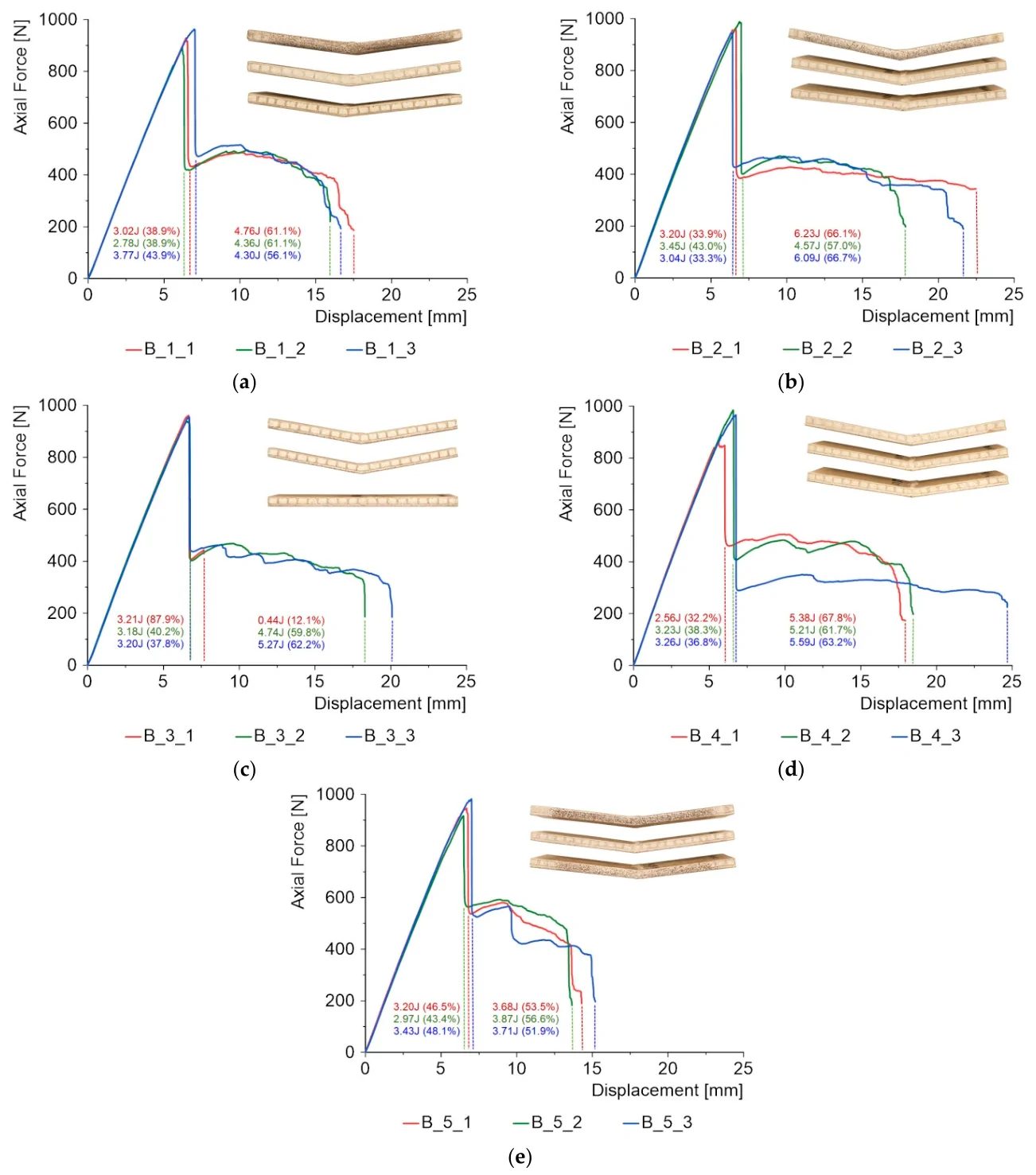
Force–displacement curves for the PC-integrated sandwich specimens subjected to bending: (**a**) batch B1; (**b**) batch B2; (**c**) batch B3; (**d**) batch B4; (**e**) batch B5.

The three-point bending results are presented in Figure 16, along with photographs of all specimens, which underwent permanent deformation due to internal structural damage. As in [33], no delamination of the PC layer was observed, which is a major advantage of this material.

The force–displacement curves followed a similar course for all investigated variants and exhibited a two-stage response.

**Figure 17:**
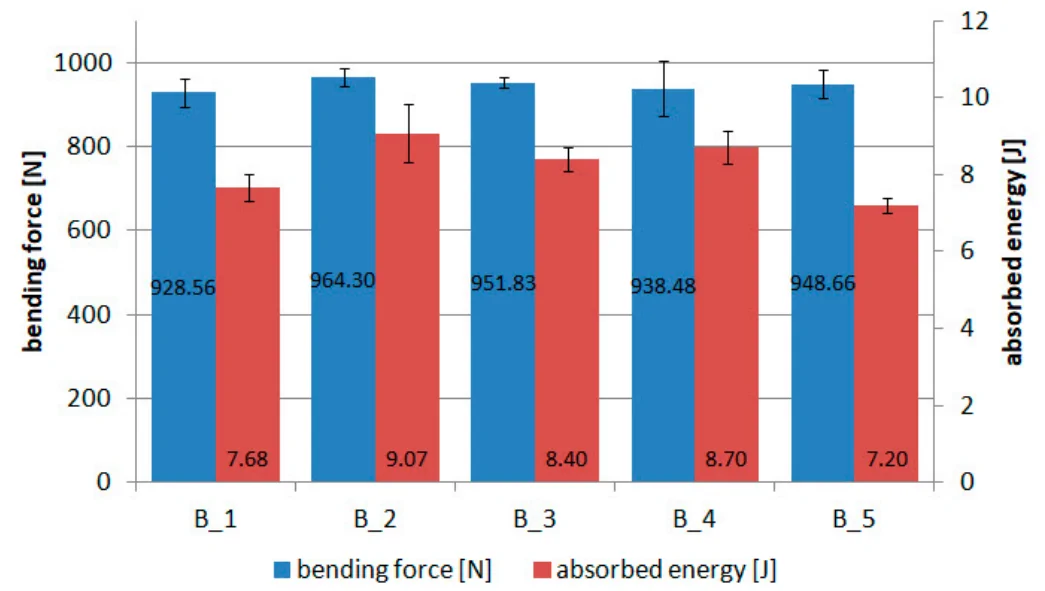
Bending-test results for the sandwich-composite specimens with pre-impregnated coatings.

**Figure 19:**
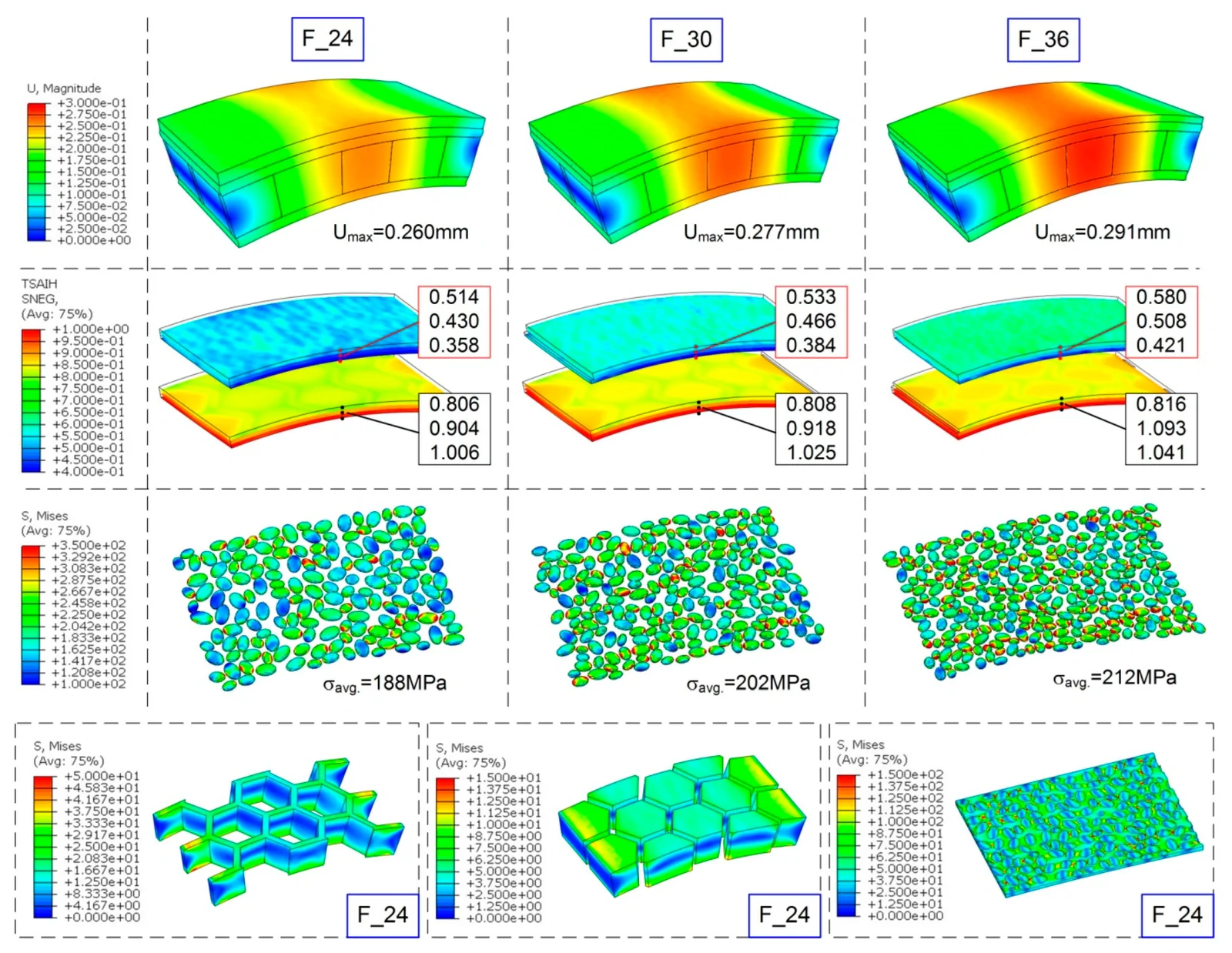
Bending-simulation results for sandwich composites with pre-impregnated coatings.

In the first stage, the force increased almost linearly to approximately 0.93–0.96 kN. This was followed by a sudden load drop to approximately 0.4–0.6 kN, after which the specimens retained substantial residual load-bearing capacity. This behaviour results from the sandwich architecture, in which the two laminate skins are not equally loaded, as demonstrated by the numerical simulation results in Figure 19. The difference in utilisation between the skins reached almost 50%. Consequently, only one skin failed initially, and a high residual capacity remained. During the second stage, corresponding to damage evolution, the force decreased progressively with increasing displacement. The duration of this process varied between specimen series and generally ended at a central displacement of 15–20 mm. Such large displacements were associated with substantial energy absorption; Figure 17, therefore, reports the energy values for both the first and second stages for each specimen. In almost every case, most of the absorbed energy was associated with the second stage of damage evolution, during which both the matrix and the reinforcement were damaged through fibre fracture and pull-out.

The maximum forces varied only slightly, from 928.56 N for series B_1 to 964.30 N for series B_2; the difference between the extreme values was therefore less than 4%. This indicates that the different PC variants did not substantially alter the initial stiffness or maximum load-bearing capacity of the overall sandwich structure.

The maximum-force and total absorbed-energy results, including error bars, are presented in Figure 17. The differences in mean absorbed energy were greater than those in maximum force, with values ranging from 7.20 to 9.07 J. The highest value was obtained for batch B_2 and the lowest for batch B_5. The maximum difference in mean absorbed energy between the investigated batches was approximately 26%, whereas the maximum-force differences remained below 4%. This indicates that the main differences between the complete PC systems occurred during the post-initiation stage of the bending response rather than in the maximum load-bearing capacity. However, crack length, crack-path evolution and local delamination were not quantified in the present study. Moreover, powder material, grit size, grain morphology and coating geometry varied simultaneously between batches. Therefore, the present results do not permit a statistical correlation to be drawn between absorbed energy, crack-propagation behaviour, and powder-grain morphology. Such a relationship would require controlled specimens with independently varied grain morphology together with quantitative crack-propagation measurements.

The percentage relative standard deviation of the mean maximum force ranged from 1.25% for batch B_3 to 7.00% for batch B_4. The mean absorbed energy ranged from 2.85% for batch B_5 to 8.20% for batch B_2. No single batch can therefore be identified as the most favourable in terms of repeatability of results. Nevertheless, no batch exceeded 10%, indicating good repeatability of the mechanical response despite the heterogeneous powder-layer architecture and possible local differences in grain distribution and resin infiltration. The observed scatter also confirms the stability of the PC manufacturing process and its integration with the sandwich composite.

### 4.3. Numerical Simulations of the RVE Models

**Figure 18:**
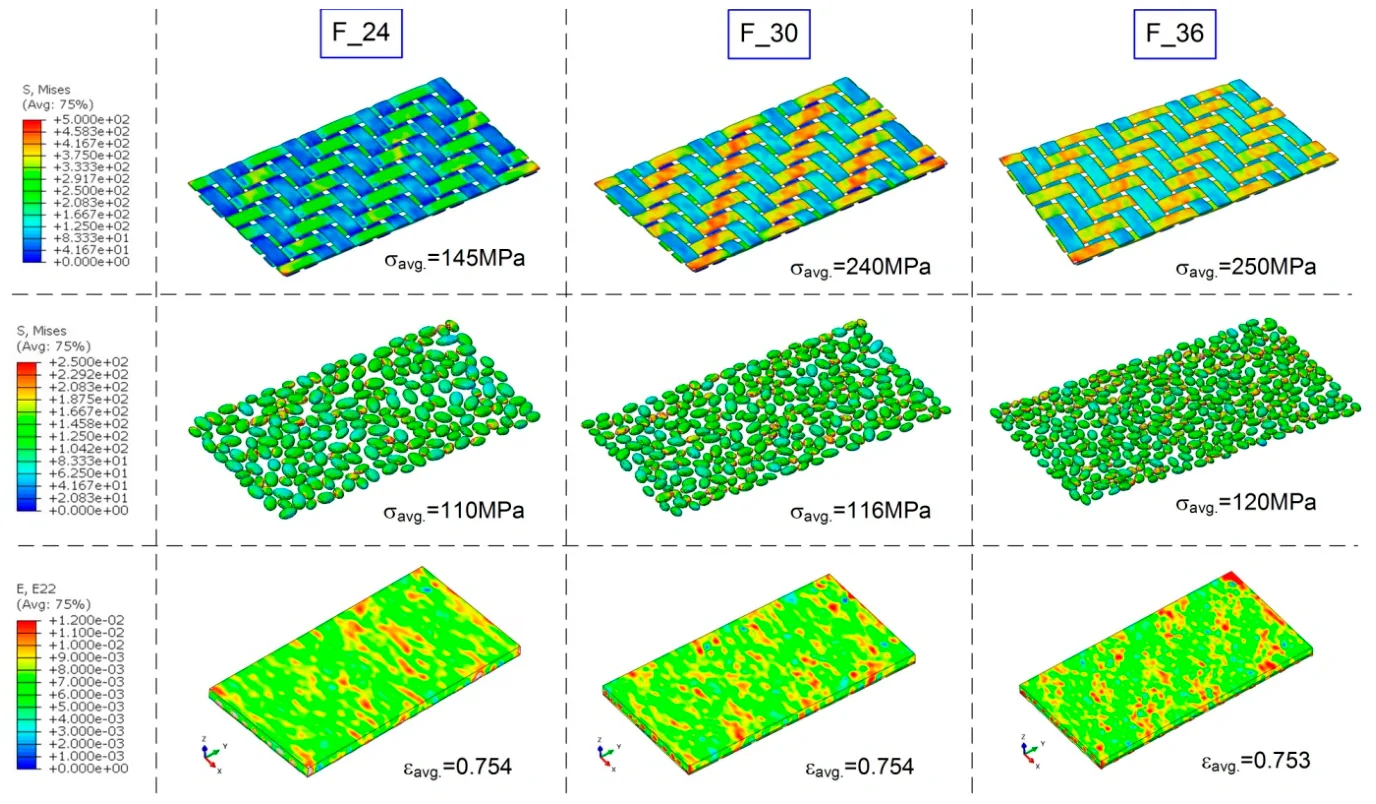
Tensile simulation results for the pre-impregnated coatings.

The results for the tensile PC RVEs shown in Figure 18 indicate that reducing the grain dimensions, and thereby increasing the number of grains within the analysed area, affected load sharing among the fabric, grains, and the thermoplastic matrix. The mean von Mises stress in the fabric tows increased from 145 MPa for F24 to 240 MPa for F30 and 250 MPa for F36. The increase in the grains was smaller, from approximately 110 to 120 MPa. At the same time, the mean transverse strain in the thermoplastic matrix remained almost unchanged.

The models, therefore, confirm that changing the grit size can substantially modify local stress fields even when the amount of thermoplastic remains unchanged. The principal qualitative agreement between the models and the experimental DIC maps lies in the heterogeneous character of the strain fields. In both the tests and simulations, local bands of elevated values were associated with the fabric paths and the vicinity of grains. The main objective of the numerical simulations was to assess the feasibility of constructing relatively large CAD and FE models. The models, however, did not reproduce the initial specimen curvature, the actual voids, the variation in fabric impregnation, or the local delamination between materials. These effects will be investigated in future work.

In the bending models, the maximum displacement increased progressively from 0.260 mm for F24 to 0.277 mm for F30 and 0.291 mm for F36 (Figure 19). The difference between the extreme cases was approximately 12%. At the same time, the mean stress in the grains increased from 188 to 212 MPa. The results indicate that grit size had a moderate effect on the global deformation of the analysed fragment but a more pronounced effect on local stresses in the functional layer. These trends refer specifically to the controlled numerical system with identical grain material properties and should not be directly assigned to the individual experimental powder batches.

The numerical comparison is restricted to the pre-peak response. The models indicate differences in global deformation and local stress transfer among the investigated grit-size variants, but they cannot reproduce stiffness degradation, crack propagation, delamination or the post-peak response observed experimentally. The Tsai–Hill criterion approached unity in some laminate plies and locally exceeded it slightly. Thus, the moment corresponding to a force of 800 N was close to the damage-initiation level in the most highly loaded regions. Because neither stiffness degradation nor damage propagation was included in the model, values above unity should be interpreted solely as indicators of potential initiation rather than as simulations of actual fracture. This interpretation is consistent with the experiment because 800 N was already relatively close to the maximum force of approximately 930–960 N.

Because Soric SF is a porous core material, the von Mises stress was not used for direct comparison with the manufacturer-reported strength values. Instead, the individual normal and shear stress components were evaluated. The calculated normal-stress ranges in the core were −9.39 to 7.72 MPa for S11, −39.07 to 31.01 MPa for S22, and −27.67 to 22.97 MPa for S33. Considering the 3-direction as the through-thickness direction of the sandwich core, the maximum transverse tensile stress was approximately 22.97 MPa, and the maximum transverse compressive stress was approximately 27.67 MPa. These values exceed the manufacturer-reported transverse tensile strength of approximately 6 MPa and compressive strength of approximately 4 MPa, respectively.

The corresponding shear-stress ranges were −6.01 to 5.84 MPa for S12, −5.02 to 5.04 MPa for S13, and −27.80 to 26.78 MPa for S23. The maximum absolute transverse shear stresses were therefore approximately 5.04 MPa for S13 and 27.80 MPa for S23, compared with the manufacturer-reported shear strength of approximately 6 MPa. These results indicate that local regions of the Soric core may reach or exceed the onset of tensile, compressive or shear damage at the applied load of 800 N. However, because material damage and stiffness degradation were not included in the FE model, these local stress exceedances should be interpreted only as indicators of potential damage initiation and not as predictions of progressive core failure.

The comparison with the experimental results is therefore intentionally restricted to the pre-peak response. The present models can be used to compare local stress, strain and displacement fields and to identify regions approaching damage initiation, but they cannot reproduce stiffness degradation, crack propagation, interfacial delamination or the post-peak force–displacement response. Consequently, the experimentally measured absorbed energy should not be quantitatively compared with the numerical results.

## 5. Conclusions

A new method for manufacturing a pre-impregnated coating was presented. The coating can be used in various composite manufacturing processes, including vacuum infusion, conventional curing, and autoclave processing. PCs can be produced using a wide range of powders and grit sizes. Their principal advantages are flexibility, which allows them to be applied to curved surfaces; the ability to cut the material to the required shape; effectively unlimited storage time owing to the thermoplastic matrix; and relatively low cost. Detailed DIC-assisted tests were conducted to determine the elastic and strength characteristics of the PCs, and the new material was successfully integrated onto the surface of a sandwich composite. The following conclusions can be drawn:

- The investigated PC systems exhibited substantial differences in tensile load-bearing capacity, with maximum forces ranging from 1.26 to 1.98 kN. Because powder material, grit size, and other coating characteristics varied simultaneously across batches, these differences cannot be attributed independently to either powder type or grit size.
- For the reference specimens, reducing the thermoplastic content from two nonwoven layers to one reduced the maximum tensile force from 2.36 to 1.95 kN, confirming the strong influence of matrix amount on the fabric-supported response.
- The PC type had little effect on the maximum bending force of the sandwich composites, which ranged from 928.56 N for batch B_1 to 964.30 N for batch B_2. Larger differences were observed in absorbed energy, which ranged from 7.20 J for batch B_5 to 9.07 J for batch B_2.
- The original Python algorithm, using the CadQuery library, successfully generated powder layers with different grit sizes and grain shapes that resembled those observed in actual powders.
- The FE models showed that reducing the grain dimensions and increasing the grain count increased the mean stresses in the fabric and grains and moderately increased the compliance of the sandwich model. Agreement with the experiments was qualitative because the models did not include initial curvature, actual defects, material non-linearity or damage propagation.
- Future work will investigate the effect of grit size for a constant powder material, examine in greater detail the degree to which the individual PC layers are filled by the thermoplastic matrix, and develop a numerical model incorporating stiffness degradation in both the PC and sandwich composite to analyse damage evolution.

## Figures and Tables

**Table 1:** Image-based particle-size characteristics, density and morphology of the powders used in the pre-impregnated coatings.

Batch	Powder	Grit Designation	D_10_ [μm]	D_10_ [μm]	D_10_ [μm]	Density [g/cm^3^]	Observed Morphology
A_1	Green silicon carbide	F220	34	54	87	3.21	angular to very angular; sharp-edged, irregular/faceted
A_2	Glauconite	F60	143	232	411	2.50	heterogeneous; predominantly subangular to subrounded, with irregular fragments and fine particles
A_3	Stainless-steel grit	DELTA 050	192	354	584	7.80	angular to very angular; sharp-edged, irregular, frequently elongated
A_4	Quartz sand	F80	94	181	419	2.65	predominantly subrounded to rounded; locally subangular
A_5	Chromium electrocorundum	F36	375	540	711	3.95	angular to very angular; strongly faceted/blocky, sharp-edged

**Table 2:** Geometrical and constituent characteristics of the reference specimens and pre-impregnated coatings.

Batch	Thickness [mm]	Areal Density [g/m^2^]	Thermoplastic Areal Mass [g/m^2^]	Powder Areal Mass [g/m^2^]	Thermoplastic Mass Fraction [wt.%]	Apparent Powder Volume Fraction [vol.%]
R1	0.28	280	-	-	-	-
R2	0.30	438	158	-	36.1	-
R3	0.30	438	158	-	36.1	-
R4	0.30	359	79	-	22.0	-
A1	0.47	642	158	204	24.6	13.5
A2	0.72	742	158	304	21.3	16.9
A3	0.65	871	158	433	18.1	8.5
A4	1.19	1136	158	698	13.9	22.1
A5	1.05	1198	158	760	13.2	18.3

**Table 3:** Post-infusion thicknesses of the B-series sandwich specimens measured from cross-sectional microscope images (mean ± SD, n = 10 measurement locations).

Batch	PC Thickness t_PC_ [mm]	Upper GFRP Skin t_u_ [mm]	Lower GFRP Skin t_l_ [mm]	Total Thickness [mm]
B_1	0.23 ± 0.12	0.95 ± 0.05	0.88 ± 0.11	4.43 ± 0.06
B_2	0.57 ± 0.13	0.89 ± 0.18	0.94 ± 0.16	4.74 ± 0.05
B_3	0.48 ± 0.12	1.14 ± 0.16	0.86 ± 0.12	4.87 ± 0.05
B_4	0.85 ± 0.09	0.85 ± 0.14	0.94 ± 0.12	5.09 ± 0.05
B_5	0.83 ± 0.09	0.76 ± 0.11	0.71 ± 0.18	5.03 ± 0.06

**Table 4:** Repeatability of the stochastic CadQuery grain-generation procedure for three independent geometrical realisations.

Grit Size	Parameter	Realisation 1	Realisation 2	Realisation 3	Mean ± SD
F24	Number of grains	181	179	177	179 ± 2
	Grain volume [mm^3^]	38.19	37.73	38.76	38.23 ± 0.52
	Grain surface area [mm^2^]	343.29	339.61	345.59	342.83 ± 3.02
F30	Number of grains	265	262	263	263.3 ± 1.5
	Grain volume [mm^3^]	32.52	32.44	32.88	32.61 ± 0.23
	Grain surface area [mm^2^]	350.13	348.49	352.4	350.34 ± 1.96
F36	Number of grains	365	367	363	365 ± 2
	Grain volume [mm^3^]	27.47	27.99	27.43	27.63 ± 0.31
	Grain surface area [mm^2^]	349.14	352.82	348.19	350.05 ± 2.45

The variability between the independent realisations was small. The relative standard deviation did not exceed approximately 1.2% for the grain count, 1.4% for the total grain volume and 0.9% for the total grain surface area. These results indicate that the global geometrical characteristics generated by the algorithm are only weakly dependent on the particular random realisation.

**Table 5:** Mechanical properties of the materials [44,45,46,47].

Material	E_x_ [GPa]	E_y_ [GPa]	E_z_ [GPa]	G_xy_ [GPa]	G_yz_ [GPa]	G_xz_ [GPa]	ν_xy_ [-]	ν_yz_ [-]	ν_xz_ [-]
grains	95	95	95	44	44	44	0.08	0.08	0.08
thermoplastic matrix	2.95	2.95	2.95	1.05	1.05	1.05	0.4	0.4	0.4
glass-fibre roving	52	15	15	5.47	5.47	5.47	0.274	0.274	0.274
GFRP	18	18	6	3.5	2.8	2.8	0.14	0.3	0.3
polyester resin	3.5	3.5	3.5	1.3	1.3	1.3	0.35	0.35	0.35
Soric SF foam core	0.85	0.85	0.85	0.32	0.32	0.32	0.33	0.33	0.33

**Table 6:** Mean equivalent radii of the initial out-of-plane deformation of the A-series tensile specimens determined from STL surface models.

Batch	Specimen 1 [mm]	Specimen 2 [mm]	Specimen 3 [mm]	Series Mean ± SD [mm]
A_1	56.7	55.8	51.9	54.8 ± 2.6
A_2	58.9	65.2	63.4	62.5 ± 3.3
A_3	38.5	39.1	38.3	38.6 ± 0.4
A_4	40.7	41.7	40.7	41.0 ± 0.6
A_5	119.5	162.1	110.2	130.6 ± 27.7

## Data Availability

The original contributions presented in this study are included in the article. Further inquiries can be directed to the corresponding author.

## Abbreviations

The following abbreviations are used in this manuscript:

PC	pre-impregnated coating
FRP	fibre-reinforced polymer
DIC	digital image correlation
RVE	representative volume element
GFRP	glass fibre-reinforced polymer
CFRP	carbon fibre-reinforced polymer
PVD	physical vapour deposition
UHMWPE	ultra-high-molecular-weight polyethylene
TBC	thermal barrier coating
PMC	polymer-matrix composite
7YSZ	7% yttria-stabilised zirconia
PEEK	polyetheretherketone
YAG	yttrium aluminium garnet
VARTM	vacuum-assisted resin transfer moulding
FEPA	Federation of European Producers of Abrasives
MEKP	methyl ethyl ketone peroxide
BL	backing layer
FL	functional layer
CNC	computer numerical control
FE	finite element
CAD	computer-aided design
STEP	Standard for the Exchange of Product Model Data
IGES	Initial Graphics Exchange Specification
CAE	computer-aided engineering
STL	stereolithography (surface-mesh file format)
SD	standard deviation
RSD	relative standard deviation
